# Integrated framework for designing durable green corrosion inhibitors from *Spirulina*: Cultivation, extraction, and electrochemical evaluation

**DOI:** 10.1016/j.isci.2026.115220

**Published:** 2026-03-04

**Authors:** Ana Fonseca, Lizeth Gutierrez Pua, Alejandra M. Miranda, Fabian Hernandez-Tenorio, Alex A. Sáez, Yaneth Pineda Triana

**Affiliations:** 1 Department of Mechanical Engineering, Universidad del Norte, Barranquilla 080001, Colombia; 2 Biosciences and Technology Research Group (Techlife), School of Applied Sciences and Engineering, Universidad EAFIT, Medellín 050022, Colombia; 3 School of Applied Sciences and Engineering, Universidad EAFIT, Medellin 050022, Colombia; 4 Department of Metallurgical Engineering, Universidad Pedagógica y Tecnológica de Colombia, Tunja 150003, Colombia

**Keywords:** Electrochemistry, Corrosion, Biomass

## Abstract

This study presents a comprehensive framework for designing eco-friendly corrosion inhibitors from *Spirulina platensis*. Biomass production was optimized for yield and quality, followed by selective extraction using ultrasound-assisted and orbital-shaking methods applied to wet and dry biomass. Extracts were chemically characterized and tested via potentiodynamic polarization and electrochemical impedance spectroscopy. The dry-biomass ultrasound-assisted extract (D1) showed the highest inhibition efficiency (90%–92%) and achieved a corrosion rate of ∼1.41 mm ${\text{year}}^{-1}$ at 1,000 ppm, maintaining ∼77% efficiency over 72 h. Statistical analysis confirmed that the presence of *Spirulina* extracts significantly improved corrosion resistance, with extract type, concentration, and immersion time identified as key variables. This work validates the potential of microalgal formulations as effective green inhibitors and offers a reproducible methodology for the long-term evaluation of bio-based corrosion protection strategies relevant to sustainable materials engineering.

## Highlights

• Optimized Spirulina cultivation yields balanced biochemical composition
• Dry biomass + ultrasound extraction maximizes inhibitor efficiency
• D1 achieved 92% inhibition and CR = 1.41 mm/year at 1,000 ppm
• Time-dependent EIS confirmed durable protection (90%–77% over 72 h)

## Graphical abstract

**Figure:**
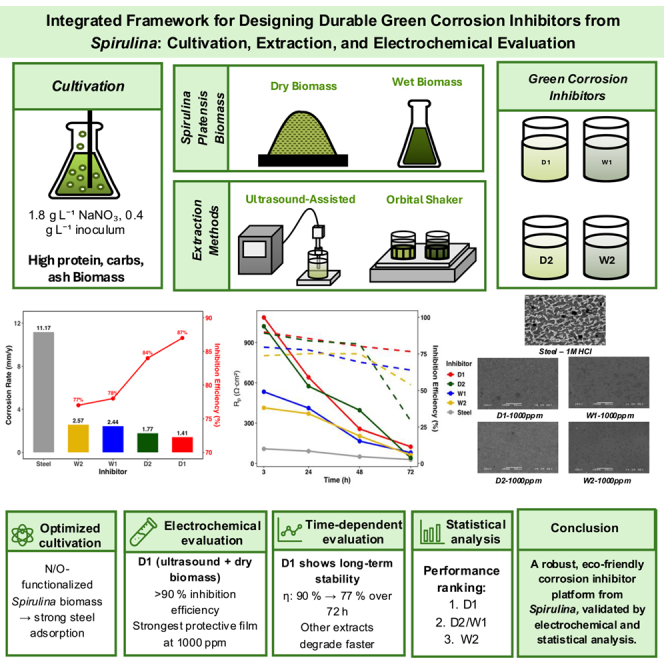

## Introduction

Corrosion remains a major economic and operational challenge in the oil and gas industry, with annual global costs exceeding $2.5 trillion—equivalent to 3%–4% of global GDP.^1^^,^^2^ Within this sector, recent estimates place corrosion-related losses at over $60 billion globally and approximately $27 billion in the United States, with U.S. upstream operations alone spending $1.372 billion per year on corrosion management in pipelines and downhole tubing.^3^ These figures exclude indirect costs from downtime, leaks, and safety incidents.^4^ Corrosion leads to equipment degradation, production losses, and increased risk, particularly under harsh conditions involving elevated temperature, pressure, or acidic treatments such as pickling and acidizing.^5^ To mitigate these effects, corrosion inhibitors are commonly introduced into corrosive media; however, conventional inhibitors, often based on amines, chromates, or phosphates, pose environmental and health risks due to their toxicity.^6^^,^^7^

In response to the limitations of conventional inhibitors, microbial extracellular polymeric substances (EPSs) are increasingly recognized as sustainable alternatives for corrosion control due to their eco-friendly nature, biodegradability, and functional versatility.^8^^,^^9^^,^^10^ EPSs are high molecular weight biopolymers, primarily polysaccharides and proteins, secreted by microorganisms including bacteria, fungi, archaea, and algae.^8^^,^^11^ These compounds can form protective films on metal surfaces, chelate metal ions, and alter interfacial electrochemical properties, thus hindering corrosion processes.^8^^,^^9^ Among EPS-producing organisms, photosynthetic microalgae have emerged as particularly attractive due to their high biomass productivity, flexible and scalable cultivation conditions, and rich biochemical composition, including antioxidant proteins and polysaccharides with potential inhibitory activity.^8^^,^^12^^,^^13^

Several microalgal species have demonstrated high inhibition efficiencies in acidic environments. For instance, *Spirulina maxima* biomass achieved 96.4% inhibition after 72 h of immersion in 1 M HCl at 100 mg ${\text{L}}^{-1}$,^14^ while *Chlorella sorokiniana* reached 94.6% under similar conditions but at 24 h.^15^ Solvent-derived extracts from *Chlorella vulgaris*, tested on API 5L 42 steel, provided 88.2% and 91.2% efficiency at 120 ppm in 1 M HCl.^16^ Likewise, a commercial *Spirulina* powder, rich in antioxidant compounds, showed 96% inhibition at 600 ppm after 12 h of immersion, as measured by weight loss at 303 K.^17^

Despite these encouraging results, several gaps remain. Most studies emphasize inhibition efficiency at single time points, without examining how upstream variables, such as cultivation conditions, biomass state (wet vs. dry), or extraction method, affect the chemical composition and functional performance of the extracts. Adsorption mechanisms at the metal interface are rarely explored at the molecular level, and the long-term stability of inhibition under extended exposure is often overlooked.^18^^,^^19^ Moreover, although extraction techniques such as solvent reflux, orbital shaking, and ultrasound-assisted extraction are known to influence phytochemical content,^20^^,^^21^ systematic comparisons of their impact on corrosion inhibition remain scarce. Time-dependent effects such as film degradation or desorption are also seldom addressed, despite experimental evidence suggesting that inhibition efficiency can decline over time.^22^^,^^23^

To address these gaps, this study develops an integrated framework for producing, evaluating, and validating green corrosion inhibitors derived from *Spirulina platensis*. We first optimized microalgal cultivation to obtain high-quality biomass, followed by selective extraction using ultrasound-assisted and orbital-shaking methods on both wet and dry biomass. The resulting extracts were chemically characterized and tested using potentiodynamic polarization and electrochemical impedance spectroscopy (EIS), including analysis of the interfacial relaxation time constant. Inhibition efficiency was assessed over 72 h, and statistical analysis was applied to determine the influence of extract type, concentration, and immersion time. Our results contribute a scalable, statistically validated methodology for developing sustainable corrosion inhibitors with potential applications in environmentally responsible industrial practices.

## Results

### *Spirulina platensis* biomass characterization

#### Moderate nitrogen levels (1.8 g/L) maximize biomass productivity by minimizing salinity-induced stress

**Figure 1:**
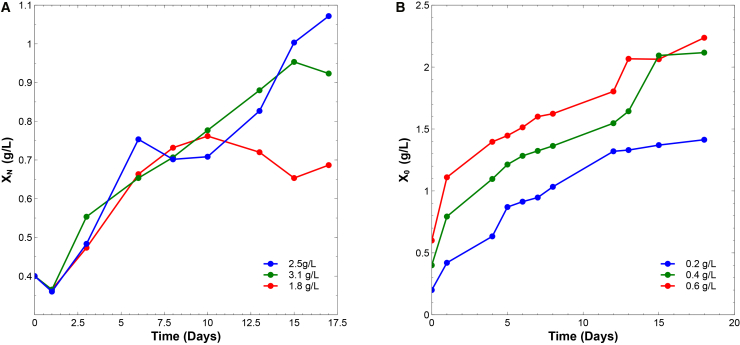
Optimization of nitrogen and inoculum levels enhances biomass accumulation in *Spirulina platensis* (A) Effect of sodium nitrate (${\text{NaNO}}_{3}$) concentration on biomass productivity. Yield peaked at 1.8 g L^−1^; higher concentrations negatively affected growth, likely due to osmotic or metabolic stress. (B) Effect of initial inoculum concentration on biomass accumulation. An inoculum of 0.4 g L^−1^ resulted in maximal yield, while 0.6 g L^−1^ reduced efficiency due to self-shading and light limitation.

The results in Figure 1A show that sodium nitrate (${\text{NaNO}}_{3}$) concentration strongly influences the growth of *Spirulina platensis*. The condition with 1.8 g/L ${\text{NaNO}}_{3}$ yielded the highest biomass in the shortest cultivation time, likely due to efficient nitrogen assimilation and a moderate metabolic stimulus.^24^ In contrast, increasing the concentration to 3.1 g/L did not improve productivity, suggesting other limiting factors, such as light or inorganic carbon availability.^25^^,^^26^

High nitrate levels may also increase medium salinity, triggering osmotic and oxidative stress that impairs photosynthetic efficiency, particularly photosystem II (PSII).^27^^,^^28^ These findings indicate that 1.8 g/L ${\text{NaNO}}_{3}$ represents an optimal nitrogen supply that maximizes biomass productivity without inducing physiological stress.^26^

#### Optimal inoculum density (0.4 g/L) enhances growth by balancing light availability and nutrient use

In the pilot-scale cultivation stage (Figure 1B), initial biomass concentration $\left(X_{0}\right)$ was a key determinant of productivity. Cultures started at 0.4 g/L achieved the highest final biomass, likely due to optimal light penetration and efficient nutrient uptake. Higher inocula (0.6 g/L) exhibited reduced growth, attributed to self-shading and early nutrient depletion.^29^

Specific productivities were 0.040, 0.031, and 0.017 g/L⋅day for $X_{0}$ values of 0.2, 0.4, and 0.6 g/L, respectively. One-way ANOVA confirmed significant differences (p<0.05), with Tukey’s HSD identifying the 0.4 g/L condition as optimal. Interestingly, high nitrogen supply reduced productivity, possibly due to the diversion of metabolic fluxes toward carbohydrate and lipid synthesis, increasing energetic demands.^30^^,^^31^

These trends align with previous studies reporting that intermediate cell densities improve both nutrient assimilation and light utilization efficiency in microalgal cultures.^32^^,^^33^^,^^34^

#### *Spirulina* biomass under optimal conditions contains proteins, polysaccharides, and minerals relevant for corrosion inhibition

To assess the biochemical profile of the biomass obtained, proximate analyses were conducted across all cultivation conditions. Emphasis was placed on the most productive condition (1.8 g/L ${\text{NaNO}}_{3}$ and 0.4 g/L $X_{0}$), which yielded a well-balanced composition typical of *Spirulina* strains and promising for corrosion inhibition applications (Table 1).

**Table 1:** Proximate composition of *Spirulina platensis* biomass under optimized cultivation conditions (1.8 g L${\;}^{-1}$${\text{NaNO}}_{3}$ and 0.4 g L${\;}^{-1}\;X_{0}$)

Parameter	Content (%)
Proteins	38
Carbohydrates	20
Moisture	12
Ash	15
Fiber	10
Fats	5

The high protein content (38%) suggests an abundance of amino acids and peptides bearing functional groups (amines, carboxyls, and amides) that may adsorb onto metal surfaces.^35^^,^^36^ Additionally, carbohydrate and fiber fractions (30% total) provide polysaccharides capable of forming compact, cohesive films on steel, acting as physical barriers.^37^^,^^38^ The mineral (ash) content may further contribute to surface passivation via oxide or mineral film formation.^39^^,^^40^

These compositional features position the biomass as a viable natural feedstock for sustainable corrosion inhibitor formulations.^41^^,^^42^^,^^43^^,^^44^

### Inhibitor characterization

#### Protein- and polysaccharide-rich functional groups dominate the chemical signature of *Spirulina* extracts

**Figure 2:**
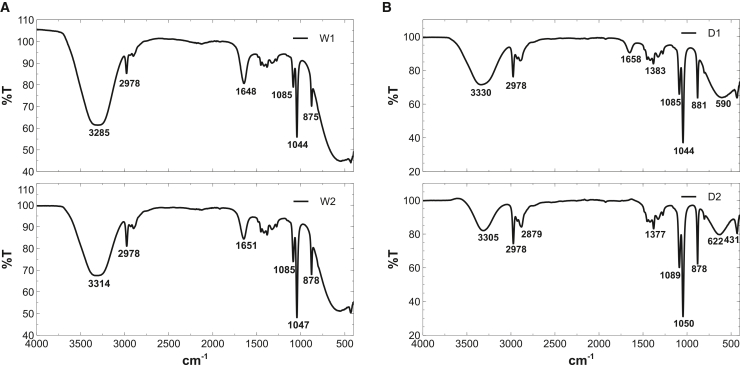
FTIR spectra of *Spirulina platensis* extracts (A) Wet-biomass extracts (W1 and W2). (B) Dry-biomass extracts (D1 and D2) exhibit characteristic bands of hydroxyl and amine groups (3,285–3,330 ${\text{cm}}^{-1}$), aliphatic C–H stretching (2,978–2,879 ${\text{cm}}^{-1}$), carbonyl/amide I vibrations (1,640–1,658 ${\text{cm}}^{-1}$), and C–O/C–N stretching (1,080–1,040 ${\text{cm}}^{-1}$). These signals confirm the presence of phenolic, protein, lipid, and polysaccharide fractions, which contribute active sites for adsorption and underpin the corrosion-inhibition behavior of the extracts. Detailed assignments are provided in Table S1.

Figure 2 presents the FTIR spectra of the *Spirulina platensis* extracts (see Table S1 for detailed band assignments). All samples exhibit a broad absorption band in the 3,285–3,330 ${\text{cm}}^{-1}$ region, attributed to O–H and/or N–H stretching vibrations associated with phenolic compounds, amino acids, and proteins, consistent with previous FTIR studies of *Spirulina* biomass.^45^^,^^46^^,^^47^^,^^48^ Among the extracts, D1 displays the most intense signal (3,330 ${\text{cm}}^{-1}$), indicating a higher abundance of hydroxylated and nitrogen-containing species.

Weak bands in the 2,978–2,879 ${\text{cm}}^{-1}$ region correspond to aliphatic C–H stretching, suggesting the presence of lipid or alkyl moieties.^46^^,^^49^ Pronounced absorptions between 1,640 and 1,658 ${\text{cm}}^{-1}$, particularly in W1, W2, and D1, are assigned to amide I vibrations of proteins and/or conjugated carbonyl groups, confirming the proteinaceous nature of the extracts.^46^^,^^50^

A distinct band near 1,383 ${\text{cm}}^{-1}$ observed exclusively in D1 may originate from symmetric methyl deformation and overlapping carboxylate vibrations, potentially reflecting contributions from protein side chains; however, due to band congestion in this region, the assignment remains tentative.^51^ All spectra show intense bands in the 1,080–1,040 ${\text{cm}}^{-1}$ range, characteristic of C–O stretching in polysaccharides and/or C–N stretching in peptide linkages.^47^^,^^52^ Additional shoulders near 900–870 ${\text{cm}}^{-1}$ have been associated with C–N vibrations linked to nitrogen heterocycles.^53^

Overall, the FTIR profiles confirm the presence of multiple oxygen- and nitrogen-bearing functional groups (O–H, N–H, C=O, C–O, and C–N) derived from proteins, polysaccharides, and phenolic fractions. These functionalities provide potential adsorption sites for interaction with steel surfaces and underpin the corrosion inhibition behavior of the extracts.

#### Ultrasound-assisted extraction of dry biomass yields protein-rich extracts with enhanced antioxidant activity

**Figure 3:**
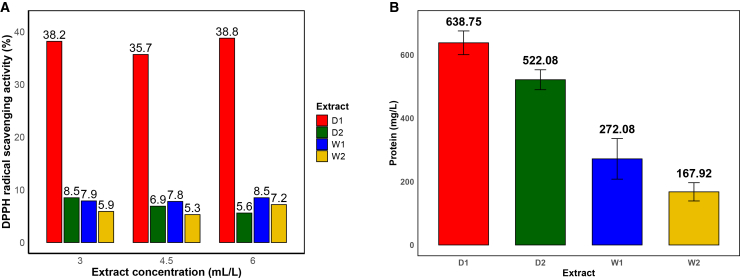
Extraction strategy governs antioxidant activity and protein recovery (A) DPPH radical scavenging activity of wet- (W1 and W2) and dry-biomass (D1 and D2) extracts at different concentrations. (B) Soluble protein content determined by the Lowry method, showing significantly higher protein levels in dry-biomass extracts (D1 and D2) compared to wet-biomass extracts (W1 and W2). Data are represented as mean ± SD (*n* = 3). Statistical differences were analyzed using one-way ANOVA followed by Tukey’s post hoc test (*p* < 0.05).

The DPPH radical scavenging assay (Figure 3A) revealed marked differences in antioxidant capacity among the extracts. D1 consistently exhibited the highest activity, exceeding 35% inhibition across all tested concentrations, indicating a stable and concentration-independent antioxidant response. This behavior suggests a higher abundance of hydrogen-donating bioactive compounds, likely associated with proteinaceous and phenolic constituents.^54^^,^^55^ In contrast, W1, W2, and D2 showed substantially lower activities (5%–9%) with no clear concentration dependence.

Soluble protein quantification (Figure 3B) further highlights the impact of extraction strategy. Dry-biomass extracts displayed significantly higher protein concentrations, with D1 and D2 reaching 638.75 and 522.08 mg ${\text{L}}^{-1}$, respectively, compared to 272.08 mg ${\text{L}}^{-1}$ (W1) and 167.92 mg ${\text{L}}^{-1}$ (W2). The enrichment of nitrogen-containing compounds in dry-biomass extracts supports the FTIR observations and suggests a greater density of functional groups capable of donating electrons and forming coordinated interactions with steel surfaces.^56^^,^^57^

Taken together, these results demonstrate that extraction methodology plays a decisive role in defining the biochemical and functional properties of *Spirulina*-derived inhibitors. Ultrasound-assisted extraction of dry biomass (D1) produces extracts enriched in proteins and redox-active compounds, which are expected to enhance adsorption and corrosion protection, whereas orbital shaking of wet biomass yields extracts with comparatively lower bioactive content.

### Electrochemical evaluation

#### Dry biomass and ultrasound-assisted extracts form more protective films as evidenced by EIS

EIS was employed to assess the anticorrosive performance of the *Spirulina* extracts. Prior to the EIS measurements, the open circuit potential (OCP) was monitored for approximately 20 min to ensure electrochemical stabilization of the electrode/electrolyte interface. During this period, the OCP showed only minor fluctuations and stabilized within a potential window of −440±5 mV, confirming that the system had reached steady-state conditions suitable for reliable impedance acquisition and minimal transient influence.^58^^,^^59^ All EIS experiments were subsequently conducted potentiostatically at the stabilized OCP, using a sinusoidal perturbation of 10 mV RMS. The OCP remained within the initial fluctuation range throughout the measurements.

**Figure 4:**
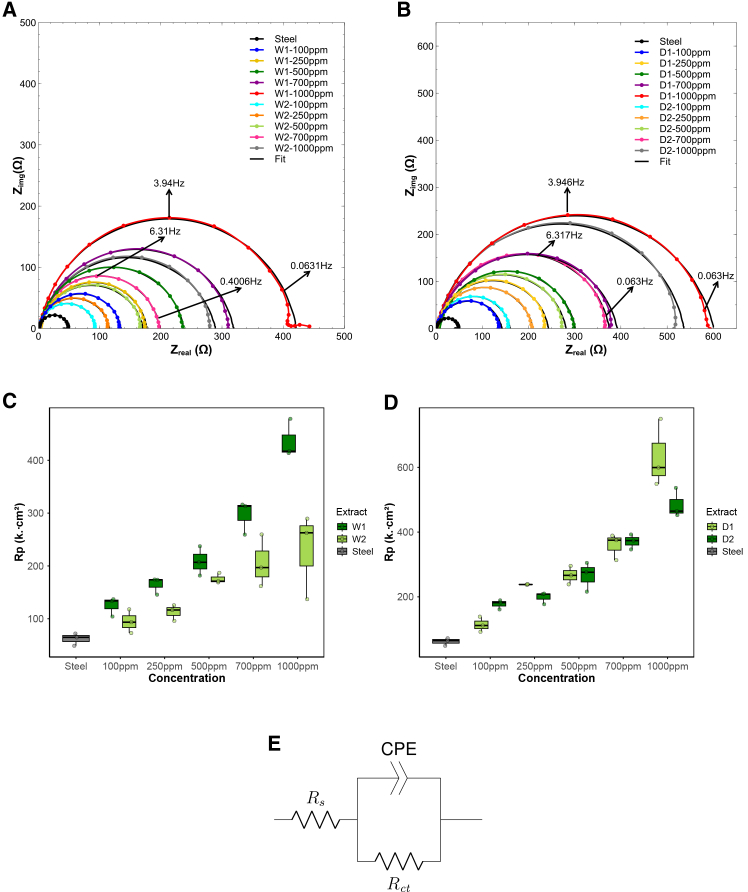
Nyquist plots for W- and D-series extracts at different concentrations and equivalent circuit model (A and B) Nyquist impedance spectra of carbon steel in 1 M HCl containing W1, W2, D1, and D2 extracts at increasing concentrations. Enlarged semicircles indicate higher charge transfer resistance and improved corrosion inhibition. (C and D) Boxplots of polarization resistance $\left(R_{p}\right)$ values for all extracts and concentrations. Boxes represent the interquartile range (IQR), center lines indicate the median, and whiskers denote minimum and maximum values (*n* = 3 per condition). (E) Equivalent electrical circuit used to fit the EIS data. Statistical differences in $R_{p}$ were analyzed using one-way ANOVA followed by Tukey’s post hoc test (p <0.05).

Nyquist plots for all extracts (Figures 4A and 4B) exhibit enlarged semicircles with increasing extract concentration, indicating improved corrosion resistance due to the formation of surface films that hinder charge transfer. The impedance spectra were fitted using the equivalent circuit shown in Figure 4E, which consists of the solution resistance $\left(R_{s}\right)$ in series with a parallel combination of charge-transfer resistance $\left(R_{ct}\right)$ and a constant phase element (CPE). The inclusion of the CPE, instead of an ideal capacitor, accounts for the non-ideal capacitive behavior of the electrochemical double layer, commonly observed in corrosion systems due to surface heterogeneity, roughness, and incomplete inhibitor adsorption.

The impedance of the CPE is given by:

(Equation 1) $$Z_{\mathrm{CPE}}=\frac{1}{Q{\left(j\omega \right)}^{n}},$$

where Q is the frequency-independent CPE constant, ω=2πf is the angular frequency, n is the empirical phase factor (0≤n≤1), and j is the imaginary unit. When n approaches 1, the CPE approximates an ideal capacitor; at n=0.5, it reflects Warburg diffusion; and at n=0, it becomes purely resistive.

The effective double-layer capacitance $\left(C_{dl}\right)$ was derived from the fitted Q, n, $R_{s}$, and $R_{ct}$ values using the Helmholtz model:

(Equation 2) $$C_{dl}=Q^{1/n}{\left(R_{s}^{-1}+R_{ct}^{-1}\right)}^{\frac{1-n}{n}}.$$

Additionally, the interfacial relaxation time constant τ—which characterizes the discharge kinetics of the electrode/electrolyte interface—was calculated as:

(Equation 3) $$\tau ={\left(Q\cdot R_{ct}\right)}^{1/n}.$$

Higher τ values are typically associated with slower charge/discharge behavior and more persistent protective films.

The fitted parameters (Table 2) consistently show that $R_{ct}$ increases and $C_{dl}$ decreases with increasing concentration, confirming the progressive adsorption of inhibitor molecules and the development of a protective interfacial layer on the steel surface. This behavior leads to effective surface blocking and impedes the charge-transfer process, in agreement with previous studies on similar inhibitor systems.^60^^,^^61^^,^^62^^,^^63^

**Table 2:** EIS parameters for steel in 1 M HCl with and without inhibitors

Inhibitor	$R_{s}$	$R_{ct}$	Q	$C_{dl}$	n	τ	$\chi^{2}$	η	$\theta_{\text{max}}$
	($\Omega \cdot {\text{cm}}^{2}$)	($\Omega \cdot {\text{cm}}^{2}$)	(S⋅${\text{s}}^{n}$${\text{cm}}^{-2}$)	(F⋅${\text{cm}}^{-2}$)		(s)		(%)	(°)
Steel + HCl 1 M	1.27	61.89	2.46E-04	4.65E-02	0.91	0.010065	1.50	–	−64.28
W1-100	1.32	125.00	2.10E-04	3.15E-02	0.89	0.016955	2.77	50	−69.3
W1-250	2.48	164.57	2.08E-04	4.25E-02	0.92	0.025135	2.80	62	−66.0
W1-500	2.05	208.67	1.48E-04	2.09E-02	0.89	0.019952	8.03	70	−70.4
W1-700	2.94	296.17	1.50E-04	2.06E-02	0.89	0.029755	1.95	79	−69.1
W1-1000	2.06	436.47	1.50E-04	2.16E-02	0.89	0.046651	0.567	86	−73.1
W2-100	0.94	94.80	2.16E-04	3.11E-02	0.91	0.013658	2.04	35	−74.08
W2-250	0.77	112.73	2.36E-04	4.19E-02	0.91	0.018505	1.82	45	−71.83
W2-500	1.33	175.63	1.70E-04	2.87E-02	0.90	0.019762	2.28	65	−68.27
W2-700	1.39	206.10	1.61E-04	2.48E-02	0.88	0.021235	1.20	70	−71.69
W2-1000	1.61	229.73	1.47E-04	2.48E-02	0.87	0.020030	1.18	73	−68.44
D1-100	2.16	114.11	1.88E-04	2.77E-02	0.89	0.013438	2.31	46	−72.0
D1-250	2.02	238.37	1.49E-04	2.65E-02	0.91	0.025080	0.971	74	−69.5
D1-500	2.94	266.93	1.37E-04	1.74E-02	0.88	0.023365	1.42	77	−62.9
D1-700	1.34	359.13	1.29E-04	1.56E-02	0.88	0.030012	1.12	83	−70.3
D1-1000	2.00	632.77	1.20E-04	1.11E-02	0.86	0.049385	1.15	90	−71.9
D2-100	1.70	177.47	1.43E-04	1.96E-02	0.89	0.016221	2.23	65	−71.32
D2-250	1.24	198.50	1.57E-04	2.26E-02	0.89	0.020238	5.30	69	−71.68
D2-500	3.20	265.83	1.63E-04	2.40E-02	0.89	0.029837	1.01	77	−64.09
D2-700	2.10	370.97	1.10E-04	1.56E-02	0.88	0.026740	0.461	83	−71.58
D2-1000	2.19	484.67	9.54E-05	1.70E-02	0.87	0.029304	2.11	87	−71.99

To further illustrate the influence of biomass state and extraction method on the interfacial response, Figure S2 (related to Figure 4A) presents Nyquist plots and box-plot summaries comparing each wet-dry pair (W1 vs. D1 and W2 vs. D2) across concentrations. In these comparative layouts, ultrasound-assisted extracts (W1 and D1) consistently exhibit larger semicircles and higher $R_{ct}$ distributions than their orbital-shaken counterparts (W2 and D2), with the dry-ultrasound combination (D1) yielding the most resistive and capacitive response. These visual trends support the statistical and quantitative observations described in Figure 4A and Table S10.

Quantitatively, W1 achieved $R_{ct}$ values up to 436 $\Omega \cdot {\text{cm}}^{2}$ with 79%–86% inhibition efficiency, compared to 206–230 $\Omega \cdot {\text{cm}}^{2}$ and 70%–73% for W2. Among the dry biomass extracts, D1 showed the highest performance ($R_{ct}=632.77$ $\Omega \cdot {\text{cm}}^{2}$, η=90%), followed by D2 (∼485 $\Omega \cdot {\text{cm}}^{2}$, η=87%).

The relaxation time constant (τ), which reflects the stability of the interfacial film,^64^ increased from 0.010 s (steel) to 0.047 s (W1) and 0.050 s (D1) at 1,000 ppm, suggesting more persistent surface coverage. In contrast, W2 and D2 plateaued at lower values (∼0.02–0.03 s), indicating less stable adsorption layers.^65^^,^^66^ Since τ is rarely reported in microalgal inhibitor studies, this metric adds novel insight into interfacial dynamics (Figure S1).

**Figure 5:**
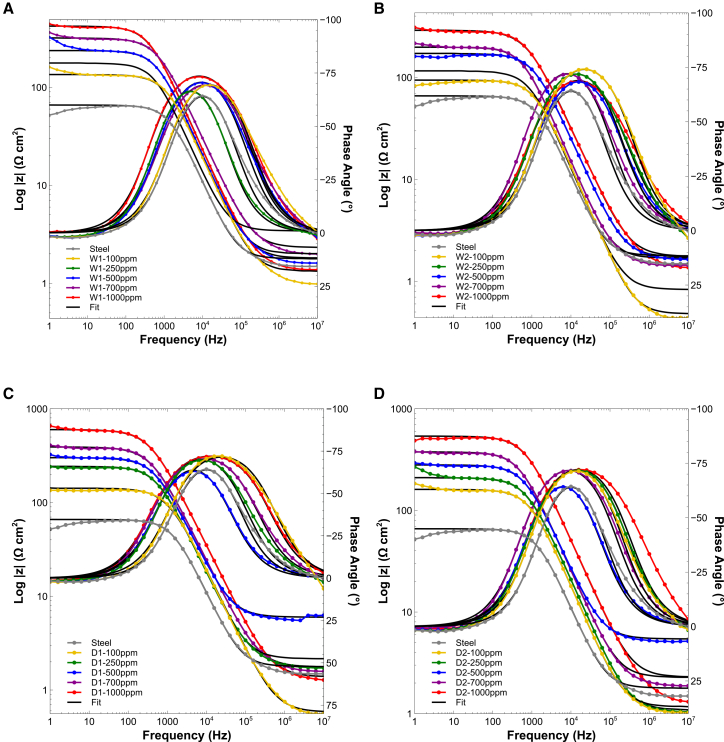
Bode plots of impedance magnitude and phase for W- and D-series extracts (A) W1, (B) W2, (C) D1, and (S) D2. Ultrasound-assisted extracts (W1 and D1) show higher log|Z| values and phase angles approaching −90°, consistent with more capacitive and compact protective films. Conversely, orbital extracts (W2 and D2) exhibit lower impedance and sharper phase peaks, indicating less uniform adsorption and weaker film formation.

Bode plots (Figure 5) further supported these observations: D1 and W1 displayed higher impedance magnitudes and phase angles approaching −90°, consistent with smooth, capacitive film formation. D2 and W2 exhibited lower log|Z| and sharper phase transitions, indicating less uniform adsorption.^64^^,^^67^^,^^68^ Boxplots of polarization resistance $\left(R_{p}\right)$ (Figures 4C and 4D) confirmed D1’s superior performance across replicates.

Statistical analysis validated these trends. One-way ANOVA showed significant effects of concentration on $R_{ct}$ for all extracts (p<0.05), with D1 (F=40.80, p<0.0001), and W1 showing the strongest dose-response relationships. two-way ANOVA confirmed significant main effects of extract type (F=53.14, p<0.0001), concentration (F=118.64, p<0.0001), and their interaction (F=7.08, p<0.0001), demonstrating that extract performance depends on both composition and dosage.

**Figure 6:**
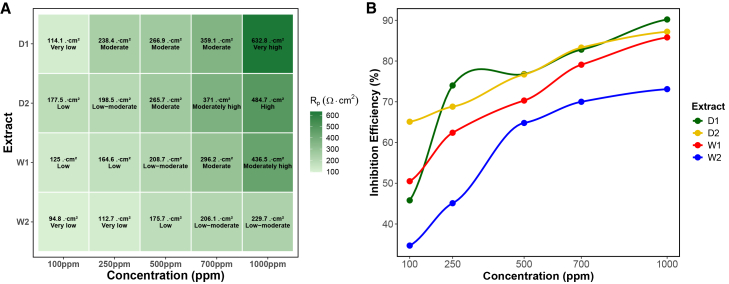
Post hoc statistical analysis of EIS results (A) Tukey heatmap of polarization resistance $\left(R_{p}\right)$ highlighting statistical groupings across extracts and concentrations, with D1 at 1,000 ppm classified as *very high resistivity* and W2 consistently in the lowest tiers. Statistical groupings were determined using one-way ANOVA followed by Tukey’s test (p < 0.05). (B) Correlation between concentration and inhibition efficiency (%IE), showing continuous improvement for D1, D2, and W1, while W2 plateaued near 70%.

Post hoc Tukey analysis stratified extracts into statistically distinct resistivity tiers (Figure 6A), with the full classification shown in Table S10. In this framework, D1 at 1,000 ppm formed the top resistivity group, followed by D2 at 1,000 ppm, whereas W2 consistently occupied the lower tiers across all concentrations. This statistical grouping highlights how both biomass state and extraction method influence corrosion inhibition performance. Correlation analysis (Figure 6B) further revealed continuous improvement in inhibition efficiency (%IE) for D1, D2, and W1 with increasing concentration, while W2 plateaued after 500 ppm, suggesting early saturation of adsorption sites.

Altogether, these results highlight that extraction method exerts a stronger influence than biomass state. The combination of ultrasound and dry biomass (D1) yields the most resistive and stable interfacial films, achieving the highest protection levels among all tested conditions.

#### Polarization curves confirm mixed-type inhibition and rank extract performance in agreement with EIS

**Figure 7:**
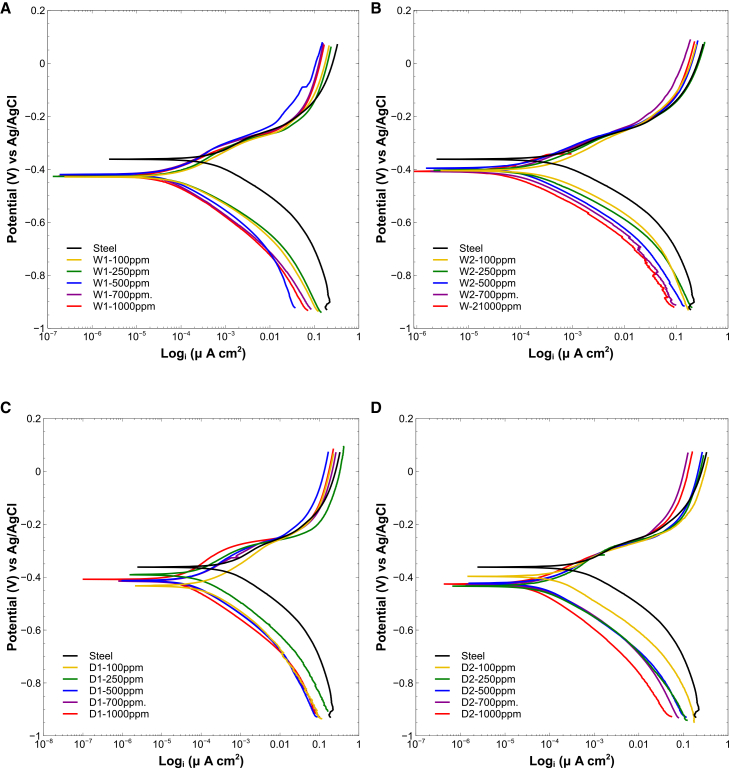
Potentiodynamic polarization (Tafel) curves for steel in 1 M HCl with and without microalgal extracts (A) W1, (B) W2, (C) D1, and (D) D2 microalgal inhibitors. All inhibitors decreased the corrosion current density $\left(I_{\text{corr}}\right)$ relative to bare steel, consistent with mixed-type inhibition. Dry-biomass extracts (D1 and D2) achieved the highest efficiencies, while wet-biomass extracts (W1 and W2) also provided substantial protection, confirming the adsorption-driven inhibition mechanism.

Potentiodynamic polarization (Figure 7) confirmed the inhibitory behavior observed in EIS. All extracts reduced corrosion current density $\left(I_{\text{corr}}\right)$ relative to bare steel, indicating mixed-type inhibition via adsorption. D1 and D2 again provided the highest protection, with D1 achieving 91.98% inhibition and a corrosion rate (CR) of 0.21 mm⋅ ${\text{y}}^{-1}$. W1 and W2 reached 90.56% and 84.64%, respectively.

The corrosion potential $\left(E_{\text{corr}}\right)$ shifted negatively, and both anodic and cathodic slopes decreased with inhibitor addition, indicating suppression of both dissolution and hydrogen evolution reactions. Tafel data (Table 3) followed the same ranking as EIS: D1 > D2 > W1 > W2.

**Table 3:** Tafel parameters for inhibitors W1, W2, D1, and D2 at various concentrations

Inhibitor	$\beta_{a}$ (mV/dec)	$\beta_{c}$ (mV/dec)	$E_{\text{corr}}$ (mV)	$I_{\text{corr}}$ (μA/cm^2^)	CR (mm/y)	η (%)
Steel + HCl 1 M	85.67	113.50	−358.3	428.0	2.4889	–
W1-100 ppm	88.80	104.13	−424.7	100.9	1.0490	76.42
W1-250 ppm	88.63	99.20	−427.7	82.0	0.6175	80.84
W1-500 ppm	83.57	107.20	−423.7	74.7	0.4514	82.55
W1-700 ppm	98.17	109.13	−419.7	57.7	0.4404	86.52
W1-1000 ppm	81.30	104.20	−419.7	40.4	0.4338	90.56
W2-100 ppm	88.83	103.27	−384.7	168.88	1.5550	60.54
W2-250 ppm	89.00	97.07	−393.7	151.00	0.6953	64.72
W2-500 ppm	81.87	98.13	−403.3	103.80	0.4830	75.75
W2-700 ppm	87.67	97.47	−405.7	99.40	0.4640	76.78
W2-1000 ppm	81.37	96.60	−409.3	65.73	0.3091	84.64
D1-100 ppm	97.37	100.79	−426.3	118.7	0.9589	76.45
D1-250 ppm	83.20	98.10	−415.3	85.17	0.6097	80.10
D1-500 ppm	93.23	101.77	−412.0	72.2	0.4571	83.13
D1-700 ppm	83.57	100.47	−421.3	62.2	0.5030	85.46
D1-1000 ppm	88.13	114.77	−415.3	34.3	0.2109	91.98
D2-100 ppm	98.83	106.97	−420.0	98.4	0.8691	77.01
D2-250 ppm	97.57	105.60	−428.7	82.27	0.5926	80.78
D2-500 ppm	82.67	104.50	−424.7	72.67	0.5091	83.02
D2-700 ppm	89.60	102.47	−424.7	64.17	0.4333	85.01
D2-1000 ppm	81.23	116.77	−424.0	53.37	0.3731	87.53

Taken together, EIS and polarization measurements demonstrate that the corrosion inhibition performance of *Spirulina* extracts is governed by concentration and extraction strategy. Ultrasound-assisted extracts from dry biomass (D1) deliver the most robust protection due to higher bioactive compound recovery and stronger surface adsorption.

#### Langmuir model best describes adsorption behavior and confirms spontaneous physisorption

**Figure 8:**
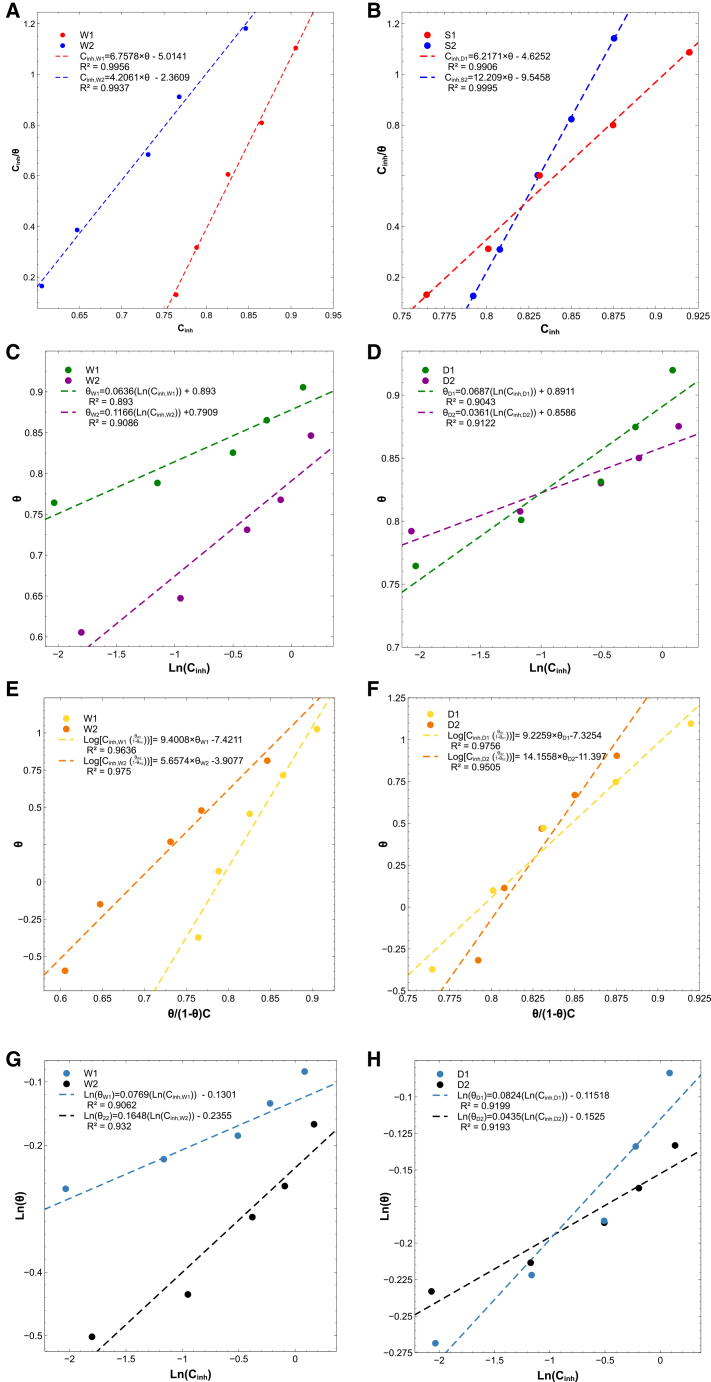
Adsorption isotherm analysis for all extracts (W1, W2, S1, and S2) (A and B) Langmuir linear plots for W1 and W2, showing excellent correlation $\left(R^{2}\right)$ and monolayer adsorption behavior. (C and D) Temkin isotherm fits indicating adsorbate-adsorbent interactions. (E and F) Frumkin isotherm plots illustrating lateral interactions among adsorbed species. (G and H) Freundlich linearizations representing non-ideal and multilayer adsorption. All isotherms were constructed from surface coverage (θ) values derived from corrosion current densities, and regression statistics are reported in Table 4.

Adsorption isotherms were constructed from the experimental coverage data to clarify how inhibitor molecules interact with the carbon steel surface. Four common models (Langmuir, Freundlich, Temkin, and Frumkin) were evaluated, and the Langmuir adsorption isotherm provided the best linear fit for all extracts, with regression coefficients $\left(R^{2}\right)$ exceeding 0.99 (see Figure 8). This result indicates that the adsorption process is dominated by monolayer formation, in which each active site on the metal surface is occupied by a single inhibitor molecule.^69^^,^^70^^,^^71^ Such monolayer coverage effectively blocks active sites and reduces anodic dissolution $\left(M\to M^{2+}+2e^{-}\right)$, thereby promoting surface passivation and inhibiting corrosion reactions.^72^^,^^73^

The equilibrium adsorption constants $\left(K_{\text{ads}}\right)$ obtained from the linear Langmuir plots were used to calculate the standard Gibbs free energy of adsorption $\left(\Delta {G{}^{\circ}}_{\text{ads}}\right)$ via:

(Equation 4) $$\Delta {G{}^{\circ}}_{\text{ads}}=-RT\ln\left(55.5\;K_{\text{ads}}\right)$$

where R is the universal gas constant (8.314 J mol^−1^ K^−1^), T is the absolute temperature (298 K), and 55.5 represents the molar concentration of water in solution. Table 4 summarizes the resulting $K_{\text{ads}}$ and $\Delta {G{}^{\circ}}_{\text{ads}}$ values for each extract.

**Table 4:** Langmuir equilibrium constants $\left(K_{\text{ads}}\right)$ and Gibbs free energies of adsorption $\left(\Delta {G{}^{\circ}}_{\text{ads}}\right)$ for microalgal extracts

Extract	$R^{2}$	$K_{\text{ads}}$ (L/mol)	$\Delta {G{}^{\circ}}_{\text{ads}}$ (kJ/mol)
W1	0.9956	$1.48\times 10^{-4}$	−21.85
W2	0.9937	$2.38\times 10^{-4}$	−20.67
S1	0.9906	$1.61\times 10^{-4}$	−21.64
S2	0.9995	$8.19\times 10^{-5}$	−23.31

According to the literature, $\Delta {G{}^{\circ}}_{\text{ads}}$ values can be used to differentiate adsorption mechanisms. Values less negative than approximately −20 kJ/mol are typically associated with physisorption, dominated by electrostatic attraction or van der Waals forces between the inhibitor molecules and the metal surface,^69^^,^^74^ whereas values more negative than about −40 kJ/mol suggest chemisorption, involving charge sharing or transfer and possible coordinate bond formation.^75^^,^^76^^,^^77^ The negative $\Delta {G{}^{\circ}}_{\text{ads}}$ values obtained for all extracts (ranging from −20.67 to −23.31 kJ/mol) confirm that adsorption is a spontaneous, thermodynamically favorable process. Furthermore, being in the intermediate range around −20 to −24kJ/mol indicates that physisorption is the predominant adsorption mechanism, likely driven by electrostatic interactions between polar functional groups in the extracts (as evidenced by FTIR) and the charged steel surface.

Among the extracts, S2 showed the most negative $\Delta {G{}^{\circ}}_{\text{ads}}$, implying comparatively stronger adsorption affinity despite its relatively smaller $K_{\text{ads}}$. This suggests that, while all extracts form spontaneous adsorbed layers, subtle differences in molecular composition can modulate the strength and stability of the adsorption process.

These findings, together with the electrochemical and spectroscopic results, support a corrosion inhibition mechanism predominantly governed by spontaneous physisorption. The progressive increase in $R_{ct}$ and corresponding decrease in $C_{dl}$ with concentration (Table 2) indicate the formation of adsorbed protective layers that hinder charge transfer. The moderate negative values of $\Delta {G{}^{\circ}}_{\text{ads}}$ (ranging from −20.67 to −23.31 kJ/mol) further confirm the physical nature of the adsorption process. Additionally, FTIR analysis revealed the presence of polar functional groups (e.g., hydroxyl, carboxyl, and amino) capable of interacting electrostatically with charged metal sites. Altogether, these results suggest that the extracts form protective monolayers on the steel surface via weak, non-covalent interactions that reduce anodic dissolution and improve corrosion resistance.^69^^,^^72^^,^^77^

#### Dry-biomass extracts exhibit superior inhibition in immersion testing

**Figure 9:**
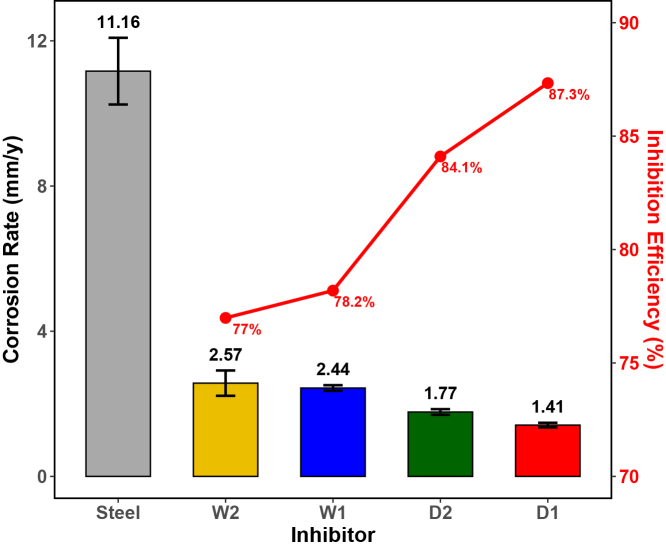
Gravimetric analysis of CR for steel in 1 M HCl with and without inhibitors CR values for bare steel and steel treated with W1, W2, D1, and D2 extracts after 24 h immersion. Bars represent mean ± SD (*n* = 3). The red line corresponds to inhibition efficiency (IE%) calculated from gravimetric measurements. All extracts significantly reduced the CR relative to bare steel (11.17 mm ${\text{y}}^{-1}$). Dry-biomass extracts (D1 and D2) achieved the highest efficiencies (87%–84%), while wet-biomass extracts (W1 and W2) also provided notable protection (77%–78%). Statistical differences were analyzed using one-way ANOVA followed by Tukey’s post hoc test (p < 0.05).

Gravimetric immersion tests were performed at 1,000 ppm, the most effective concentration based on EIS data, to validate long-term inhibition performance under ASTM G31 conditions.^78^ As shown in Figure 9, all *Spirulina*-based formulations significantly reduced the CR of steel in 1 M HCl compared to the uninhibited control.

Bare steel exhibited a CR of 11.17 mm⋅ ${\text{y}}^{-1}$ after 24 h of immersion. In contrast, all microalgal extracts reduced the CR to below 3 mm⋅ ${\text{y}}^{-1}$, demonstrating efficient suppression of metal dissolution over extended exposure. D1 and D2 extracts were the most effective, lowering the CR to 1.41 and 1.77 mm⋅ ${\text{y}}^{-1}$, with corresponding inhibition efficiencies (IE%) of 87% and 84%. W1 and W2, derived from wet biomass, achieved slightly lower inhibition (CR = 2.44 and 2.57 mm⋅ ${\text{y}}^{-1}$; IE% = 78% and 77%).

These findings are consistent with prior reports on microalgae-based green inhibitors. For example, *Chlorella sorokiniana* extracts reached 94.6% IE in 1 M HCl after 24 h,^15^ while *Spirulina maxima* demonstrated 82.6% efficiency at 100 mg⋅ ${\text{L}}^{-1}$ (CR = 1.18 × 10^−3^ g⋅ cm^−2^⋅ h^−1^).^14^ *Chlorella vulgaris* extracts have reported CR values of 4.28–5.72 mm⋅ ${\text{y}}^{-1}$, with IE up to 91.1%,^16^ while *Chlorococcum* sp. extracts containing levoglucosenone and hexadecanoic acid showed strong inhibition at 15.6 ppm (CR ≈ 2.5 mm⋅ ${\text{y}}^{-1}$).^79^ Marine strains such as *Synechococcus* sp. and *Thalassiosira* sp. have also achieved IE% 64%–78% in saline media after 192–384 h,^80^ underscoring the robustness of algal inhibitors across species and exposure times.

Statistical evaluation confirmed the superiority of D-series extracts. One-way ANOVA revealed significant differences in both CR and IE% across treatments (p<0.001). Post hoc LSD analysis (Table S11) stratified the samples into statistically distinct groups: D1 consistently ranked highest, followed by D2, while W1 and W2 formed a moderate-performance tier. These results confirm the enhanced protective effect of dry-biomass extracts, especially those obtained via ultrasound-assisted extraction, which likely promote higher release and surface adsorption of active biomolecules.

#### Surface protection correlates with biomass state and extraction method

**Figure 10:**
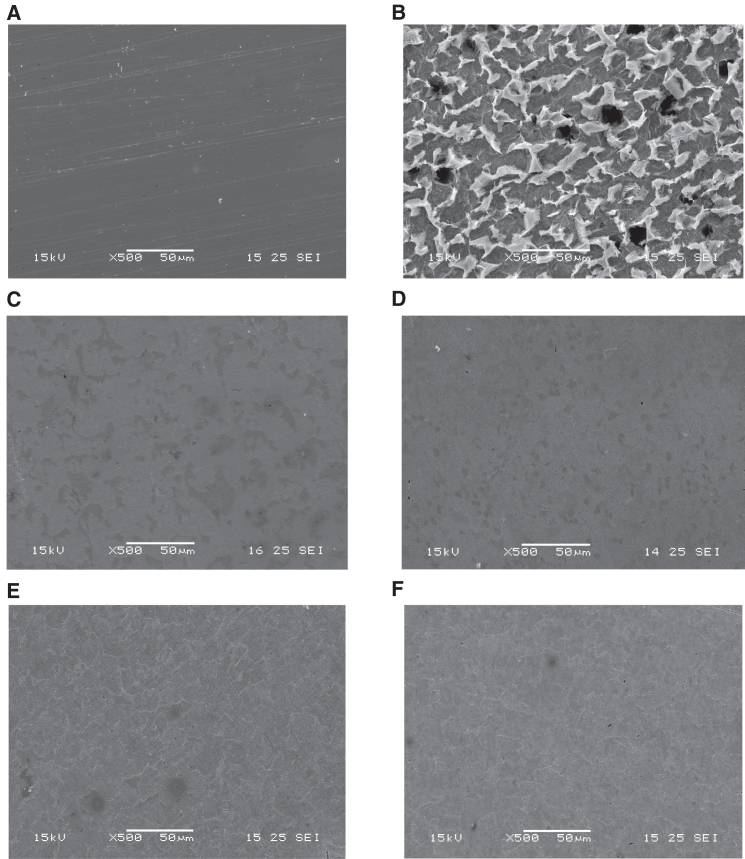
SEM micrographs of steel surfaces after 24 h immersion in 1 M HCl with and without inhibitors (A) Polished steel before immersion. (B) Steel after exposure to 1 M HCl without inhibitor. (C–F) Surfaces treated with *Spirulina*-derived extracts W1, W2, D1, and D2, respectively. Inhibited specimens exhibit smoother and more intact surface morphologies compared to the uninhibited control. Scale bars, 50 μm (A–F).

Scanning electron microscopy (SEM) was used to visually assess surface damage after 24-h immersion in 1 M HCl with and without inhibitors. As shown in Figure 10A, the untreated steel surface (Figure 10B) exhibited extensive corrosion, including deep pits and widespread degradation. This morphology confirms the aggressive nature of the acidic medium and matches the high CR observed in gravimetric tests (11.17 mm⋅ ${\text{y}}^{-1}$).

In contrast, the surfaces exposed to microalgal inhibitors (Figures 10C–10F) retained much smoother and more intact morphologies, validating their protective effect. The dry-biomass extracts D1 and D2 produced the most uniform coverage, with minimal surface roughness and no visible pitting. These results align with their superior electrochemical and gravimetric performance.

Wet-biomass extracts (W1 and W2) also reduced surface damage compared to the uninhibited control but showed less compact and continuous film formation. Some microstructural features remained exposed, suggesting incomplete surface protection.

Overall, these images confirm that *Spirulina*-based extracts mitigate corrosion by forming surface-adsorbed inhibitor films, with performance strongly dependent on biomass state and extraction method. The superior coverage observed for D1 and D2 reinforces the findings from EIS, Tafel, and immersion testing, supporting a film-forming, adsorption-driven inhibition mechanism.

#### Dry-biomass extracts suppress iron dissolution more effectively, as confirmed by UV-vis

**Figure 11:**
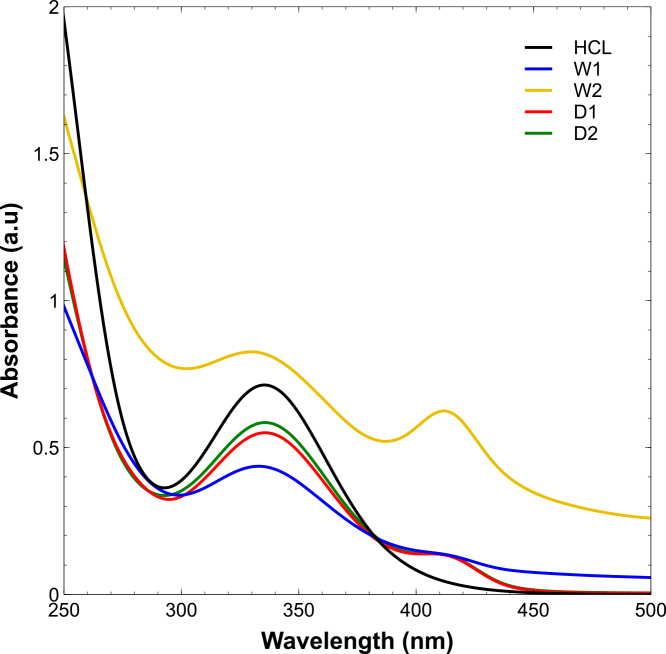
UV-visible spectra of 1 M HCl solutions after 24 h immersion with and without *Spirulina* extracts (1,000 ppm) The uninhibited solution shows strong absorbance (280–450 nm) due to soluble ${\text{Fe}}^{3+}$ corrosion products. Inhibitor-treated solutions exhibit attenuated absorbance, most pronounced for dry-biomass extracts (D1 and D2), indicating effective suppression of iron dissolution through compact surface adsorption. Wet-biomass extracts (W1 and W2) reduce absorbance less markedly, with W2 maintaining broader bands, consistent with partial film formation or soluble extract-iron complexation.

To further investigate corrosion inhibition mechanisms, UV-visible spectra were recorded for the acidic test solutions after 24 h immersion of steel coupons in 1 M HCl with or without microalgal extracts (Figure 11). The uninhibited solution displayed high absorbance between 280 and 450 nm, consistent with soluble ${\text{Fe}}^{3+}$ corrosion products,^81^ confirming active steel degradation.

In contrast, all inhibitor-containing solutions exhibited reduced absorbance in this region, indicating lower levels of dissolved iron. The suppression was most pronounced for dry-biomass extracts (D1 and D2), which showed strong attenuation in the 330–410 nm range. This suggests effective surface adsorption that limited iron dissolution and release into the electrolyte.

W1 also decreased absorbance but less markedly, consistent with partial film formation observed in SEM images. W2 maintained broader absorption bands, possibly due to (i) residual chromophoric compounds that remained in solution, (ii) ${\text{Fe}}^{3+}$-extract complex formation, or (iii) less compact adsorption on the steel surface.^82^ Despite this, W2 still achieved inhibition efficiencies above 70%, implying that solution-phase interactions also contribute to its protective effect.

These spectral results reinforce the mechanistic understanding derived from gravimetric and electrochemical measurements: dry-biomass, ultrasound-assisted extracts (especially D1) form more compact, adsorbed films that suppress both corrosion and the release of soluble products.

#### Long-term electrochemical assessment highlights stability differences among microalgal inhibitors

**Figure 12:**
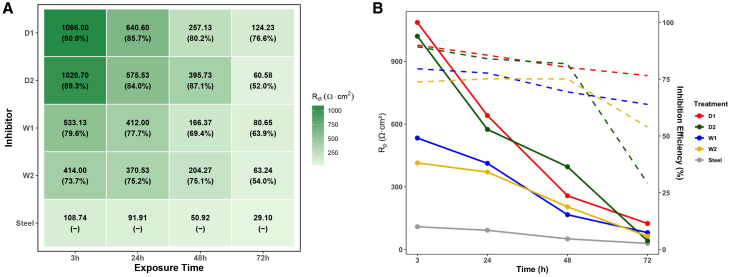
Time-dependent behavior of *Spirulina platensis* inhibitors at 1,000 ppm (A) Heatmap of charge transfer resistance $\left(R_{ct}\right)$ and inhibition efficiency (%IE) measured at 3, 24, 48, and 72 h. Color intensity reflects relative $R_{ct}$ magnitude. (B) Temporal evolution of polarization resistance ($R_{p}$, solid lines) and inhibition efficiency (%IE, dashed lines) over 72 h exposure in 1 M HCl. Data are presented as mean ± SD (*n* = 3). Statistical differences were evaluated using one-way ANOVA followed by Tukey’s post hoc test (p <0.05).

The long-term corrosion-inhibition behavior of the extracts was evaluated by EIS after 3, 24, 48, and 72 h immersion in 1 M HCl at 1,000 ppm. Figure 12 summarizes the time-dependent evolution of polarization resistance $\left(R_{p}\right)$ and inhibition efficiency (%IE). The corresponding Nyquist plots are provided in the supplemental information (Figure S3).

At 3 h, all extracts displayed high inhibition efficiencies (≥ 85%) and large Nyquist semicircles, indicative of rapid adsorption of polar biomolecules, such as proteins, pigments, and fatty acids, onto active corrosion sites. This rapid film formation is consistent with previous reports on other microalgal inhibitors, including *Tetraselmis chuii*, *Chlorella sorokiniana*, and *Scenedesmus* sp.^15^^,^^83^

By 24 h, the protective effect remained largely stable for most formulations, suggesting good persistence of the adsorbed layers. Comparable durability has been observed in extracts from *Spirulina maxima* and *Chlorococcum* sp., which also maintained efficiencies above 80% under similar conditions.^14^^,^^79^

At 48 h, a moderate decline in $R_{p}$ and %IE was recorded, with most extracts still achieving efficiencies above 75%. This trend likely reflects partial degradation of the protective film, possibly mitigated by secondary mechanisms such as metal-ligand complexation or molecular rearrangement at the interface.^84^^,^^85^

By 72 h, differences among the extracts became more pronounced. D1 remained the most effective, maintaining an inhibition efficiency of approximately 77%, while D2 showed a marked drop to 41%, suggesting instability or depletion of active components. W1 and W2 exhibited more gradual declines, ending at 64% and 54%, respectively (Figure 12).

This time-dependent loss of performance can be attributed to two main factors. First, prolonged exposure to the acidic medium may induce partial desorption or reorganization of the adsorbed film, especially in areas where microdefects progressively emerge, compromising the compactness and barrier function of the protective layer.^75^^,^^77^ Second, chemical degradation of bioactive molecules, such as proteins and pigments, may occur through hydrolysis or oxidation, reducing their capacity to maintain strong interactions with the metal surface over time.^76^^,^^84^ Nonetheless, the persistence of moderate $R_{ct}$ values and capacitive impedance behavior at 72 h indicates that the inhibition mechanism remains operative, albeit with a less cohesive protective film. This behavior aligns with previously reported degradation patterns of biogenic inhibitors under prolonged immersion.

Statistical analysis via two-way ANOVA confirmed significant effects of extract type, immersion time, and their interaction on $R_{p}$ values (*p* <0.001 for all). Post hoc Tukey analysis identified D1 as the top-performing and most stable extract throughout the tested period, followed by D2. Wet-biomass extracts (W1 and W2) exhibited lower performance and more gradual decline, whereas the uninhibited sample consistently showed the lowest $R_{p}$.

Overall, these results demonstrate that ultrasound-assisted dry-biomass extracts, particularly D1, not only deliver superior initial inhibition, but also sustain higher long-term performance by forming more robust and persistent protective layers. This outperforms the typical durability range observed for other natural corrosion inhibitors.^14^

#### Life cycle thinking reveals favorable environmental performance of Spirulina extracts

A conceptual life cycle assessment (LCT) was conducted to evaluate the environmental performance of *Spirulina platensis* extracts, focusing on four key stages: microalgal cultivation, extraction, application, and end-of-life (see Table S12). The analysis highlighted a favorable balance between functional efficiency and environmental impact for all extracts.

Microalgal cultivation contributes to ${\text{CO}}_{2}$ fixation while producing biodegradable biomass rich in proteins, polysaccharides, and phenolic compounds. During the extraction phase, ultrasound-assisted processing (D1) exhibited the best energy-to-efficiency ratio, achieving more effective concentration of bioactive fractions compared to orbital agitation methods. However, since methanol is used as a solvent in the final formulation, its environmental presence must be considered during the application and disposal stages.

At the use stage, extract D1 showed the highest corrosion inhibition performance, achieving initial efficiencies of 90%–92% and maintaining 77% after 72 h, reducing CRs to approximately 1.41 mm⋅ ${\text{year}}^{-1}$ at 1,000 ppm. Although methanol contributes to the environmental burden as a volatile organic compound (VOC), it is relatively less critical than chlorinated or aromatic solvents used in conventional inhibitor synthesis due to its rapid degradation and low environmental persistence.^86^

At end-of-life, the residual solution consists of biodegradable extract and methanol. This mixture should be handled as a non-aqueous organic waste stream. Nevertheless, its overall environmental impact is markedly lower than that of synthetic inhibitors with recalcitrant structures and toxic residues.^7^^,^^87^

Overall, the LCT analysis suggests that methanol-based microalgal formulations represent an environmentally favorable and potentially scalable alternative for industrial corrosion protection, particularly when benchmarked against traditional inhibitors with higher toxicity, lower biodegradability, and larger environmental footprints.^88^^,^^89^^,^^90^

## Discussions

This study presents an integrated strategy for developing green corrosion inhibitors based on *Spirulina platensis*, combining optimized cultivation, selective extraction, and time-resolved electrochemical evaluation. Under optimized growth conditions (1.8 ${\text{gL}}^{-1}$ ${\text{NaNO}}_{3}$, 0.4 ${\text{gL}}^{-1}$ initial biomass), Spirulina achieved high productivity and a balanced biochemical composition enriched in proteins, polysaccharides, and minerals, key contributors to surface adsorption and corrosion protection.

The extraction method proved critical: ultrasound-assisted extracts from dry biomass (D1) exhibited the highest protein content and antioxidant activity, together with a higher density of polar functional groups (O–H, N–H, C=O, and C–O), as confirmed by FTIR analysis. These characteristics translated into superior electrochemical performance. At 1,000 ppm in 1 M HCl, D1 achieved an inhibition efficiency of 91.98% and reduced the CR by 87%, outperforming other formulations in both EIS and Tafel analyses while forming compact, persistent protective films.

Beyond electrochemical metrics, statistical tools (ANOVA, Tukey post hoc, and correlation analysis) provided a robust framework for performance classification. D1 consistently ranked highest in resistivity, followed by D2 and W1, while W2 was the least effective. These trends were corroborated by SEM and UV-vis analyses, which showed reduced surface damage and lower iron dissolution in the most effective treatments.

A key contribution of this work is the time-resolved electrochemical assessment. Although all extracts initially showed high inhibition efficiencies (%IE≥85%), only D1 maintained stable protection (77% after 72 h), while the others declined more rapidly. This approach revealed critical durability differences and underscores the importance of time-dependent testing in evaluating green inhibitors.

A comparative analysis with previously reported microalgal corrosion inhibitors underscores the competitive performance of the *Spirulina platensis* extracts developed in this study (Table 5). The best-performing extract (D1) achieved 91.98% inhibition at 1,000 ppm in 1 M HCl and maintained 77% after 72 h. While *Spirulina maxima* biomass reported by Rodrigues et al.^14^ reached 96.4% at only 100 ppm, it relied on whole biomass, which may limit formulation flexibility. Similarly, *Chlorella sorokiniana* biomass achieved 94.6% inhibition at 100 ppm,^15^ confirming the effectiveness of whole-cell systems. In contrast, our study used selective extracts obtained via ultrasound-assisted extraction, enabling better control over active compound concentration, improved reproducibility, and easier downstream processing.

**Table 5:** Comparative inhibition efficiencies (%IE) of various microalgal and macroalgal extracts under different media and concentrations

Microalgae or macroalgae	Corrosive medium	Concentration	%IE	References
*Chlorella vulgaris* (methanol)	1 M HCl	120 ppm	88.22	Almanza et al.^16^
*Chlorella vulgaris* (chloroform/methanol)	1 M HCl	120 ppm	91.10	Almanza et al.^16^
*Spirulina platensis* (ethanolic)	1 M HCl	500 ppm	75.82	Kamal and Sethuraman^91^
*Spirulina platensis* (ethanolic)	1 M ${\text{H}}_{2}{\text{SO}}_{4}$	500 ppm	80.21	Kamal and Sethuraman^91^
*Hydroclathrus chathratus*	1 M HCl	500 ppm	65.28	Kamal and Sethuraman^92^
*Hydroclathrus chathratus*	1 M ${\text{H}}_{2}{\text{SO}}_{4}$	500 ppm	77.64	Kamal and Sethuraman^92^
*Kappaphycus alvarezii*	1 M HCl	500 ppm	69.33	Kamal and Sethuraman^93^
*Spirulina maxima*	1 M HCl	400 ppm	91.30	Rodrigues et al.^14^
*Spirulina maxima*	1 M HCl	800 ppm	96.40	Rodrigues et al.^14^
*Chlorella sorokiniana* biomass	1 M HCl	100 ppm	94.60	Oliveira^15^
*Chlorococcum* sp. (methanol)	1 M HCl	11.7 ppm	93.84	Rai et al.^79^
*Scenedesmus*-derived fatty acids	1 M HCl	36 ppm	95.10	Khanra et al.^83^
*Sargassum muticum*	1 M HCl	1500 ppm	96.60	Nadi et al.^94^
*Halopitys incurvus*	0.5 M ${\text{H}}_{2}{\text{SO}}_{4}$	600 ppm	85.30	Benabbouha et al.^95^
*Synechococcus* sp.	1 M f/2 medium	–	69.38	Chen et al.^80^
*Chlorella* sp.	1 M f/2 medium	–	70.80	Chen et al.^80^
*Thalassiosira* sp.	1 M f/2 medium	–	83.78	Chen et al.^80^
*Spirulina platensis* (D1)	1 M HCl	1,000 ppm	91.98	This work
*Spirulina platensis* (D1–72 h)	1 M HCl	1,000 ppm	77.00	This work

Other extract-based systems also showed high performance. For instance, *Chlorella vulgaris* extracts achieved 88.2% (methanolic) and 91.1% (chloroform:methanol) inhibition at 120 ppm,^16^ while *Chlorococcum* sp. reached 93.8% at just 11.7 ppm.^79^ These values are notable but often involve purified bioactive fractions (e.g., levoglucosenone and hexadecanoic acid) or guided theoretical optimization like DFT, which may hinder scalability and reproducibility.

Ethanolic extracts of *Spirulina platensis* studied by Kamal et al.^91^ achieved lower efficiencies (75.8%–80.2%) at 500 ppm. Macroalgae like *Hydroclathrus clathratus* and *Kappaphycus alvarezii* also showed moderate inhibition (65%–78%), while *Sargassum muticum* reached 97% at 1 g/L but required significantly higher loading. Marine microalgae such as *Synechococcus*, *Chlorella*, and *Thalassiosira* showed lower and declining inhibition (69%–84%) after 16 days in neutral medium.^80^ Overall, the D1 extract offers a practical, time-stable, and environmentally friendly alternative that performs comparably to or better than most other unpurified algal extracts. Other microalgal systems, such as the ethanolic extracts of *Spirulina platensis* studied by Kamal et al.,^91^ achieved lower efficiencies (75.8%–80.2%) at 500 ppm. Macroalgae like *Hydroclathrus clathratus* and *Kappaphycus alvarezii* also showed moderate inhibition (65%–78%), while *Sargassum muticum* reached 97% at 1 g/L, though requiring significantly higher loading.^92^^,^^93^^,^^94^ Some studies using isolated fatty acids from *Scenedesmus* or methanolic extracts from *Chlorococcum* achieved high inhibition at very low concentrations (11–36 ppm), but these typically involve compound purification or DFT-guided optimization, which may hinder scalability.^79^^,^^83^ Marine microalgae such as *Synechococcus*, *Chlorella*, and *Thalassiosira* in neutral media demonstrated lower efficiencies (69%–83%) over extended periods and showed declining performance over time.^80^

Overall, this work demonstrates that combining cultivation optimization, ultrasound-assisted extraction, and statistically validated, time-resolved analysis enables the rational design of high-performance, microalgae-derived corrosion inhibitors. The D1 extract emerges as a promising and scalable candidate, offering a durable, effective, and environmentally friendly alternative that compares favorably to other reported systems, particularly in terms of long-term inhibition and ease of formulation.

### Limitations of the study

While this study provides a robust framework for developing and evaluating *Spirulina*-based corrosion inhibitors, several limitations should be acknowledged. First, the chemical characterization of the extracts was limited to FTIR, UV-vis, protein content, and antioxidant assays. Although these techniques provide functional and compositional information, they do not resolve the molecular identity of the active constituents. Accordingly, more advanced analytical techniques, such as UPLC-MS, LC-MS, GC-MS, or NMR, are recommended to enable a deeper understanding of the specific molecules responsible for corrosion inhibition. This point was raised by one of the reviewers and has now been incorporated into this section.

Second, the study focused on short- and medium-term performance (up to 72 h) under static acidic conditions; long-term durability and performance under dynamic or industrially relevant flow conditions remain to be explored.

Additionally, the inhibitory mechanisms were inferred primarily from indirect techniques and functional group analysis; surface-specific methods such as XPS, AFM, or TEM could offer more direct evidence of film composition and adsorption modes. Finally, while statistical validation was used to distinguish extract performance, the study was limited to one type of steel and acid medium (1 M HCl), which may restrict generalizability to other alloys or environments. Future work should address these gaps to fully validate the scalability and versatility of microalgae-derived inhibitors.

## Resource availability

### Lead contact

Further information and requests for resources, data, and materials should be directed to and will be fulfilled by the lead contact, Ana Fonseca (fonsecama@uninorte.edu.co).

### Materials availability

This study did not generate new unique materials or biological samples.

### Data and code availability

• All data supporting the findings of this study are included in the main article and its supplemental information files. Additional raw experimental data (electrochemical measurements, statistical analyses, and summary tables) are available from the lead contact upon reasonable request, as these datasets form part of ongoing research and are not publicly archived at this time.
• R scripts and analysis code used for statistical tests, plotting, and post hoc analyses are available from the lead contact upon reasonable request.
• Any additional datasets, processed data tables, and analysis outputs are available upon reasonable request to the lead contact.
• No accession codes from community-endorsed repositories are required for this study.

## Acknowledgments

The authors acknowledge the INCITEMA Research Group (Instituto para la Investigación e Innovación en Ciencia y Tecnología de Materiales, INCITEMA), Universidad Pedagógica y Tecnológica de Colombia, and the Centro Argos para la Innovación (Medellín, Antioquia, Colombia) for their support and collaboration in this research.

## Author contributions

Conceptualization, A.F.; methodology, A.F.; investigation, A.F., L.G.P., A.M.M., and F.H.T.; formal analysis, A.F. and L.G.P.; validation, L.G.P., A.A.S., and Y.P.T.; visualization, L.G.P.; data curation, A.M.M. and F.H.T.; writing – original draft, A.F. and L.G.P.; writing – review and editing, A.F., L.G.P., A.A.S., and Y.P.T.; supervision, Y.P.T.

## Declaration of interests

L.G.P. is currently affiliated with The University of Texas at Dallas. However, this research was conducted independently and did not receive financial, material, or institutional support from that institution. The authors declare no competing interests.

## Declaration of generative AI and AI-assisted technologies in the writing process

During the preparation of this work, the authors used ChatGPT (OpenAI) to assist in refining the English language and improving the clarity of the text. Additionally, Consensus was used to support the identification of relevant literature during the early stages of manuscript development. After using these tools, the authors reviewed and edited all content as needed and take full responsibility for the content of the publication.

## STAR★Methods

### Key resources table

**Table:** 

REAGENT or RESOURCE	SOURCE	IDENTIFIER
**Chemicals, peptides, and recombinant proteins**		

Sodium nitrate (NaNO_3_)	Sigma-Aldrich	CAS: 7631-99-4
Methanol (99.8%)	Sigma-Aldrich	CAS: 67-56-1
2,2-Diphenyl-1-picrylhydrazyl (DPPH)	Sigma-Aldrich	CAS: 1898-66-4
Lowry protein assay reagent	Sigma-Aldrich	SKU TP0300-1 KT
Hydrochloric acid (HCl), 37%	Sigma-Aldrich	CAS: 7647-01-0

**Biological samples**		

*Spirulina platensis* strain 926	Universidad EAFIT culture collection	This paper

### Experimental model and study participant details

The microalgal strain used in this study was *Spirulina platensis* ATTC 1929, obtained from the culture collection of the Biotechnology Laboratory at the Argos Innovation Center in Medellín, Colombia. Biomass cultivation was conducted under two settings.

#### Laboratory scale

Cultures were grown in 2 L Erlenmeyer flasks containing sterilized Zarrouk medium.^96^ The cultures were maintained under a 12:12 light–dark cycle, ambient temperature (22 ± 5°C), light intensity of 55 ± 4 μmol m^−2^ s^−1^, and aeration with 3% CO_2_-enriched air.

#### Pilot scale

Cultivation was carried out in 20 L acrylic photobioreactors (PBRs) equipped with diffuser membranes for gas dispersion. A modified Zarrouk medium was used,^97^ and the PBRs operated under natural photoperiods and ambient temperature (22 ± 5°C) with 3% CO_2_-enriched synthetic air.

The sex of microalgal strains is not applicable. Cell authentication is not routinely performed for cyanobacteria; however, standard ATCC culturing protocols were followed to ensure strain identity and purity.

### Method details

#### Effect of nitrogen concentration on biomass and protein content (Laboratory scale)

A unifactorial experimental design was implemented to evaluate the influence of nitrogen availability on *Spirulina* biomass productivity and protein accumulation. The variable under study was sodium nitrate (NaNO_3_) concentration in the Zarrouk medium, with three treatments: high nitrogen (3.125 g/L, 25% above standard), standard nitrogen (2.5 g/L), and low nitrogen (1.8 g/L, 25% below standard). The initial cell concentration was 0.2 g/L. Biomass growth was monitored every 48 h using the gravimetric dry weight method. After 14 days, biomass yield and protein content were determined to assess treatment effects.

#### Effect of initial biomass concentration on productivity (pilot scale)

In the pilot-scale photobioreactors, an unifactorial experimental design was applied to study the effect of initial biomass concentration $\left(X_{0}\right)$ on overall productivity. Three $X_{0}$ levels were tested: 0.2, 0.4, and 0.6 g/L. Growth was monitored every 48 h through dry weight measurements. Final productivity and protein retention were used as evaluation parameters. The cultivation conditions were identical across treatments to isolate the effect of $X_{0}$.

#### Bromatological analysis

Bromatological analyses were conducted on the biomass samples obtained from the different pilot-scale treatments. Proteins, moisture, ash, crude fiber, and crude fat were quantified following AOAC Official Methods 990.03, 934.01, 923.03, 962.09, and 920.39, respectively.^98^^,^^99^^,^^100^^,^^101^

#### Extraction of *Spirulina* extracts

**Figure 13:**
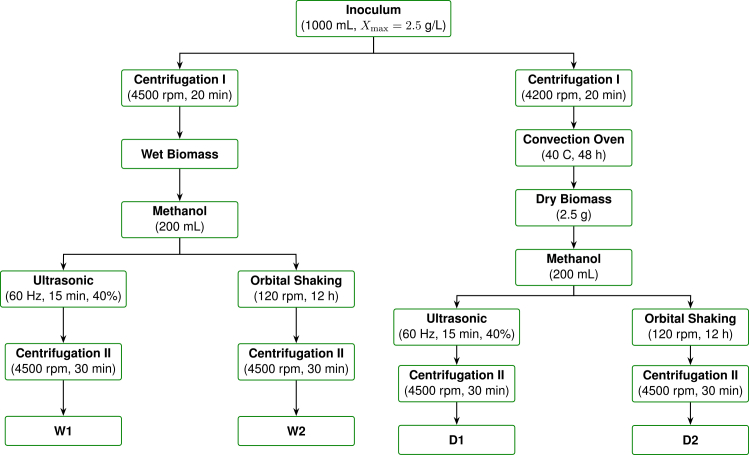
Extraction workflow for *Spirulina*-derived corrosion inhibitors Left column (wet biomass) yields W1 (ultrasonic) and W2 (orbital). Right column (dry biomass) yields D1 (ultrasonic) and D2 (orbital).

Four extracts were obtained from the pilot-scale culture with the highest productivity and protein content (1.875 g ${\text{L}}^{-1}$ ${\text{NaNO}}_{3}$, $X_{0}=0.4$ g ${\text{L}}^{-1}$; see Figure 1B). The extraction protocol was adapted from Bhagavathy et al.^102^ and Prakash et al.,^79^ with minor modifications (Figure 13). Two biomass states were considered, and each was processed with two extraction strategies.

##### Wet biomass

1000 mL aliquots of culture (strain 926, $X_{\text{max}}=2.5$ g ${\text{L}}^{-1}$) were centrifuged at 4500 rpm for 20 min. The resulting pellets were macerated in 200 mL of analytical-grade methanol for 5 min.^103^^,^^104^ Two extracts were obtained: **W1**, using ultrasound-assisted extraction, and **W2**, using orbital shaking.

##### Dry biomass

2.5 g samples were collected, centrifuged, and dried at 40 °C for 48 h to preserve thermolabile metabolites.^30^^,^^105^ Each dried sample was macerated in 200 mL methanol for 5 min. The resulting extracts were **D1** (ultrasound-assisted extraction) and **D2** (orbital shaking).

Ultrasound-assisted extraction was performed with a Branson sonicator at 60 Hz and 40% amplitude for 15 min.^49^ Orbital shaking was carried out at 120 rpm for 12 h.^106^ A final centrifugation step at 4500 rpm for 30 min was conducted in all cases to recover the supernatants, which were stored as the final extracts.

#### Functional group analysis

Functional group analysis of the microalgal extracts (D1, D2, W1, W2) was performed using Fourier Transform Infrared Spectroscopy (FTIR). A Shimadzu Prestige 21 spectrometer was used to record spectra in the range of 4500–600 ${\text{cm}}^{-1}$. Spectra were also collected for a control (uninhibited) carbon steel sample. The FTIR profiles were analyzed to identify characteristic peaks corresponding to functional groups such as proteins, polysaccharides, and phenolic compounds, which are known to contribute to corrosion inhibition through adsorption mechanisms.

#### Biochemical characterization

The biochemical composition of the four extracts was evaluated to determine the presence of antioxidant and protein-rich constituents. Antioxidant capacity was assessed using the 2,2-diphenyl-1-picrylhydrazyl (DPPH) radical scavenging assay, following established protocols.^107^^,^^108^ Soluble protein content was quantified using the Lowry method.^106^^,^^109^ These assays provided insight into the bioactive fractions of the extracts potentially responsible for corrosion protection.

#### Electrochemical impedance spectroscopy (EIS)

Corrosion behavior was evaluated using a conventional three-electrode cell. An Ag/AgCl electrode served as the reference electrode, platinum as the counter electrode, and steel specimens (exposed area of 1.2 ${\text{cm}}^{2}$) as the working electrode. The electrolyte consisted of 1 M HCl solution. EIS measurements were performed with a potentiostat/galvanostat (Gamry 750) at the open circuit potential (OCP), using a frequency range from 100 kHz to 0.01 Hz and a sinusoidal perturbation amplitude of 5 mV. All tests were conducted at room temperature and repeated in triplicate.

The experimental impedance spectra were fitted to the equivalent circuit model using *Gamry Echem Analyst*. The goodness of fit was quantified using the reduced chi-square statistic:

(Equation 5) $$\chi_{\text{red}}^{2}=\frac{1}{2N-p}\overset{N}{\underset{i=1}{\sum }}\frac{\left[{\left(Z_{i,obs}^{\prime }-Z_{i,\mathrm{fi}t}^{\prime }\right)}^{2}+{\left(Z{\prime \prime }_{i,obs}-Z{\prime \prime }_{i,\mathrm{fi}t}\right)}^{2}\right]}{\sigma_{i}^{2}}$$

where N is the number of frequency points, p the number of fitted parameters, $Z_{i,obs}^{\prime }$ and $Z{\prime \prime }_{i,obs}$ the measured real and imaginary impedance components, $Z_{i,\mathrm{fi}t}^{\prime }$ and $Z{\prime \prime }_{i,\mathrm{fi}t}$ the fitted values, and $\sigma_{i}$ the standard deviation for each data point. The uncertainty $\sigma_{i}$ was estimated as:

(Equation 6) $$\sigma_{i}=\sqrt{{\left(\alpha |Z_{i}|\right)}^{2}+\beta^{2}}$$

where α and β are proportional and constant error terms, respectively, applied equally to all spectra. The formulation of Equation 5 accounts for both the real and imaginary parts of the impedance at each frequency, as recommended for complex EIS fitting.^110^ The degrees of freedom are 2N−p since each frequency point provides two independent data components. The error model for $\sigma_{i}$, combining proportional and constant components, is commonly used to reflect instrumental noise and measurement variability.^111^^,^^112^

The inhibition efficiency based on charge transfer resistance was calculated as^63^^,^^64^:

(Equation 7) $$\eta_{\text{EIS}}\left(\%\right)=\frac{R_{ct,inh}-R_{ct,steel}}{R_{ct,inh}}\times 100$$

where $R_{ct,inh}$ and $R_{ct,steel}$ are the charge transfer resistances in the presence and absence of the inhibitor, respectively.

#### Potentiodynamic polarization (Tafel)

Potentiodynamic polarization curves were obtained in the same electrochemical cell at OCP, sweeping the potential from −0.25 V to +0.25 V vs. OCP at a scan rate of 1 mV/s. The corrosion potential $\left(E_{\text{corr}}\right)$ and corrosion current density $\left(i_{\text{corr}}\right)$ were determined by Tafel extrapolation. The inhibition efficiency based on Tafel analysis was calculated as^83^:

(Equation 8) $$\eta_{\text{Tafel}}\left(\%\right)=\frac{i_{\text{corr,steel}}-i_{\text{corr,inh}}}{i_{\text{corr,steel}}}\times 100$$

where $i_{\text{corr,steel}}$ and $i_{\text{corr,inh}}$ are the corrosion current densities in the absence and presence of the inhibitor, respectively.

#### Adsorption isotherm modeling

To evaluate the interaction between extract components and the carbon steel surface, adsorption isotherms were applied to model the surface coverage behavior as a function of inhibitor concentration. The surface coverage degree (θ) was calculated from the corrosion current densities using:

(Equation 9) $$\theta =\frac{i_{\text{corr,blank}}-i_{\text{corr,inhib}}}{i_{\text{corr,blank}}}$$

where $i_{\text{corr,blank}}$ is the corrosion current density in 1 M HCl without inhibitor, and $i_{\text{corr,inhib}}$ is the value in the presence of the inhibitor.

The following isotherm models were used to assess the adsorption behavior:

(Equation 10) $$Langmuir:\;\frac{C_{\text{inh}}}{\theta }=\frac{1}{K}+C_{\text{inh}}$$

(Equation 11) $$Freundlich:\;\ln\theta =n\ln C_{\text{inh}}+\ln K$$

(Equation 12) $$Temkin:\;\theta =-\frac{1}{2a}\ln K-\frac{1}{2a}\ln C_{\text{inh}}$$

(Equation 13) $$Frumkin:\;\frac{\theta }{1-\theta }\;e^{-2d\theta }=KC_{\text{inh}}$$

Here, $C_{\text{inh}}$ is the inhibitor concentration, K is the adsorption equilibrium constant, and n, a, and d are empirical parameters describing adsorbate–adsorbent interactions in each model. Linearization of the data was used to determine the best-fitting model and corresponding constants.

#### Gravimetric measurements

Gravimetric corrosion tests were carried out in triplicate following ASTM G31 methodology.^78^ Rectangular carbon steel coupons were immersed in 70 mL of 1 M HCl solution for 24 h at room temperature, with and without the addition of microalgal extracts (D1, D2, W1, W2). Each specimen was weighed before and after exposure using an analytical balance (± 0.1 mg). Mass loss (ΔW), corrosion rate (CR), and inhibition efficiency (η) were calculated according to Equations 14 and 15,^83^:

(Equation 14) $$CR=\frac{\Delta W\times 8.76\times 10^{4}}{A\cdot t\cdot D},$$

(Equation 15) $$\eta \left(\%\right)=\frac{CR_{without}-CR_{with}}{CR_{without}}\times 100,$$

where A is the exposed metal area (1.2 ${\text{cm}}^{2}$), t is immersion time (h), D is the steel density (g ${\text{cm}}^{-3}$), and $8.76\times 10^{4}$ is a unit conversion factor to express CR in mm ${\text{yr}}^{-1}$. Extract-free steel was used as the blank $\left(CR_{without}\right)$, and results were compared with samples containing extracts $\left(CR_{with}\right)$.

#### UV-visible spectroscopy

UV-Vis absorption spectra (200–400 nm) were recorded using a Shimadzu UV-2600 spectrophotometer in 1 M HCl to detect the possible formation of inhibitor-metal complexes after immersion of carbon steel in all the evaluated extracts.

#### Surface morphology investigation

Scanning electron microscopy (SEM) was performed on steel coupons before and after 24 h immersion in 1 M HCl with each extract (D1, D2, W1, and W2), using a Carl Zeiss EVO MA10 microscope. Morphological differences were used to visually examine the protective film formation on inhibited samples compared to severely corroded uninhibited steel.

#### Time-dependent stability of the inhibitors

Considering the operational demands of industrial environments, where metallic equipment is often exposed to aggressive media for prolonged periods, the stability and persistence of corrosion inhibitors represent critical criteria for their selection and practical application. Although the extracts investigated here are still at the development stage, it is essential to assess their time-dependent stability as corrosion inhibitors.

Accordingly, the inhibitory performance was evaluated at different exposure times in order to determine their ability to provide sustained protection. Electrochemical impedance spectroscopy (EIS) measurements were conducted on carbon steel immersed in 1 M HCl with and without each extract (1000 ppm) after immersion periods of 3, 24, 48, and 72 h. The same three-electrode cell configuration, measurement settings, and analysis procedures described in the EIS subsection were used throughout these experiments.

#### Life Cycle Thinking analysis of the microalgal inhibitor system

A conceptual life cycle analysis was conducted following a Life Cycle Thinking (LCT) approach to evaluate the sustainability of corrosion inhibitors derived from *Spirulina platensis*.^88^^,^^113^^,^^114^ The assessment considered four main stages: (i) microalgal biomass production, (ii) processing through ultrasound-assisted extraction and orbital agitation using methanol as the solvent, (iii) use phase, including performance evaluation as a corrosion inhibitor, and (iv) end-of-life stage, associated with extract biodegradability and hazardous waste management.

### Quantification and statistical analysis

All experiments were conducted in triplicate (*n* = 3), and results are reported as mean ± standard deviation (SD). The coefficient of variation (CV%) was calculated to evaluate reproducibility of electrochemical measurements. Descriptive statistics, including mean, SD, SE, CV%, and 95% confidence intervals, are provided in the Supplementary Information (Tables S2–S5 for EIS; Tables S6–S9 for Tafel analysis).

Prior to hypothesis testing, assumptions for parametric analysis were evaluated. Normality of residuals was assessed using the Shapiro–Wilk test, homogeneity of variances using Levene’s test, and independence of residuals using the Durbin–Watson statistic.

For cultivation assays (nitrogen concentration and initial biomass), one-way ANOVA (α=0.05) was applied to test differences in productivity and protein content among treatments. When significant effects were detected, Tukey’s Honest Significant Difference (HSD) test was used for multiple comparisons.

For electrochemical and gravimetric data, one-way ANOVA was applied to charge-transfer resistance $\left(R_{\text{ct}}\right)$, polarization resistance $\left(R_{p}\right)$, and corrosion rate (CR) to evaluate treatment effects. two-way ANOVA was used to assess the main and interaction effects of extract type (W1, W2, D1, D2) and concentration. Post hoc comparisons were conducted using Tukey HSD (p < 0.05). Statistical groupings presented in figures correspond to Tukey multiple comparison results.

All statistical analyses were performed using STATGRAPHICS Centurion XVI (Statgraphics Technologies Inc., USA) and RStudio version 4.2.2 (R Core Team, Vienna, Austria).
