# Supplementary material for: Integrated framework for designing durable green corrosion inhibitors from Spirulina: Cultivation, extraction, and electrochemical evaluation
Source: iScience. 2026 Mar 4;29(4):115220. doi: 10.1016/j.isci.2026.115220 (PMC13018981; doi:10.1016/j.isci.2026.115220)
Supplement: Document S1. Figures S1–S3 and Tables S1–S12 [file mmc1.pdf]

iScience, Volume *29*

Supplemental information

Integrated framework for designing durable green corrosion inhibitors from *Spirulina*: Cultivation, extraction, and electrochemical evaluation

Ana Fonseca, Lizeth Gutierrez Pua, Alejandra M. Miranda, Fabian Hernandez-Tenorio, Alex A. Sáez, and Yaneth Pineda Triana

SUPPLEMENTAL INFORMATION INDEX

Table S1. Fourier-transform infrared (FTIR) functional group assignments for *Spirulina platensis* extracts (W1, W2, D1, D2), related to Figure 2. This table summarizes the major absorption bands observed in the FTIR spectra of each extract, the tentative functional group assignments, and the associated biomolecular origins (e.g., proteins, lipids, carbohydrates, phenolics). Variations in band intensity and presence across extracts reflect differences in chemical composition linked to biomass state and extraction method. Relevant literature references supporting band assignments are included.

Band range (cm^{-1})	W1	W2	D1	D2	Functional group	Biomolecular origin	Ref.
3300–3280	3285	3314	3330	3305	O–H/N–H stretching	Phenolics, alcohols, amino acids, proteins	1–5
2980–2870	2978	2978	2978	2978, 2879	C–H stretching of CH_3/CH_2	Lipids and alkanes	1,2,5,6
1658–1640	1648	1651	1658	–	C=O stretching	Amide I, carbonyls; proteins, flavonoids	1,3,7
1380–1370	–	–	1383	1377	C–H deformation	Carbohydrate/lipid skeletal vibrations	4,7–9
1080–1040	1085	1085	1085	1089	C–O stretching	Polysaccharides, carbohydrates	1,6,7
1050–1016	1044	1047	1044	1050	C–O–C glycosidic stretch	Polysaccharides	6,7
880–870	875	878, 881	881	878	Out-of-plane =C–H bending	Aromatics, unsaturated lipids	1,3

Table S2. Descriptive statistics of impedance modulus values obtained from EIS for the steel control and W1 extracts at different concentrations, related to Figure 4a. Summary statistics include mean, standard deviation (SD), coefficient of variation (CV%), standard error (SE), and 95% confidence intervals (CI) calculated using the Student's t distribution ($n = 3$). These values provide insight into the variability and reliability of the modulus measurements across replicates.

Extract	Concentration	n	Mean	SD	CV%	SE	$t_{0.975}$	IC_inf	IC_sup
Steel	Steel	3	61.89	11.76	19.01	6.79	4.30	32.55	91.23
W1	100 ppm	3	125.0	18.10	14.48	10.45	4.30	80.07	169.93
W1	250 ppm	3	164.9	16.46	9.98	9.50	4.30	123.96	205.90
W1	500 ppm	3	208.6	27.80	13.33	16.05	4.30	139.98	277.32
W1	700 ppm	3	296.2	31.97	10.79	18.46	4.30	217.21	375.12
W1	1000 ppm	3	435.8	36.38	8.35	21.01	4.30	345.95	525.58

Table S3. Descriptive statistics of impedance modulus values obtained from EIS for the steel control and W2 extracts at different concentrations, related to Figure 4a. Summary statistics include the mean, standard deviation (SD), coefficient of variation (CV%), standard error (SE), and 95% confidence intervals (CI) calculated using the Student's t distribution for three replicates ($n = 3$). These statistics provide a measure of the variability and reliability of the impedance modulus measurements.

Extract	Concentration	n	Mean	SD	CV%	SE	$t_{0.975}$	IC_inf	IC_sup
Steel	Steel	3	61.89	11.76	19.01	6.79	4.30	32.55	91.23
W2	100 ppm	3	94.77	22.48	23.72	12.98	4.30	38.90	150.65
W2	250 ppm	3	112.7	15.23	13.51	8.79	4.30	74.98	150.36
W2	500 ppm	3	175.6	9.84	5.60	5.68	4.30	151.11	200.15
W2	700 ppm	3	206.1	49.47	24.01	28.57	4.30	82.99	329.21
W2	1000 ppm	3	229.7	81.28	35.37	46.94	4.30	27.71	431.69

Table S4. Descriptive statistics of impedance modulus values obtained from EIS for the steel control and D1 extracts at varying concentrations, related to Figure 4b. Summary statistics include the mean, standard deviation (SD), coefficient of variation (CV%), standard error (SE), and 95% confidence intervals (CI) based on triplicate measurements ($n = 3$). These values provide insight into the variability and precision of the impedance modulus measurements for each condition.

Extract	Concentration	n	Mean	SD	CV%	SE	$t_{0.975}$	IC_inf	IC_sup
Steel	Steel	3	61.89	11.76	19.01	6.79	4.30	32.55	91.23
D1	100 ppm	3	114.1	23.38	20.49	13.50	4.30	55.98	172.29
D1	250 ppm	3	238.4	0.91	0.38	0.52	4.30	235.99	240.81
D1	500 ppm	3	266.9	28.41	10.64	16.39	4.30	196.38	337.49
D1	700 ppm	3	359.1	40.21	11.20	23.21	4.30	259.18	459.03
D1	1000 ppm	3	632.8	104.9	16.58	60.30	4.30	373.21	892.28

Table S5. Descriptive statistics of impedance modulus values from EIS experiments using the steel substrate and D2 extracts at different inhibitor concentrations, related to Figure 4b. Summary statistics presented include the mean, standard deviation (SD), coefficient of variation (CV%), standard error (SE), and 95% confidence intervals (CI) calculated using the Student's t distribution for triplicate measurements ($n = 3$). These statistics highlight the reproducibility and dispersion of the modulus values across conditions.

Extract	Concentration	n	Mean	SD	CV%	SE	$t_{0.975}$	IC_inf	IC_sup
Steel	Steel	3	61.89	11.76	19.01	6.79	4.30	32.55	91.23
D2	100 ppm	3	177.5	14.53	8.18	8.39	4.30	141.11	213.89
D2	250 ppm	3	198.5	18.48	9.30	10.67	4.30	152.79	244.21
D2	500 ppm	3	265.8	45.42	17.09	26.22	4.30	153.00	378.66
D2	700 ppm	3	371.0	23.25	6.26	13.42	4.30	312.73	429.27
D2	1000 ppm	3	484.7	45.42	9.36	26.22	4.30	372.23	597.11

Table S6. Tafel summary statistics for extract W1, related to Figure 7a. Summary statistics include the mean corrosion current density ($\bar{I}_{\text{corr}}$) and corrosion potential ($\bar{E}_{\text{corr}}$), as well as the standard deviation (SD), standard error (SE), 95% confidence intervals (CI), and coefficient of variation (CV%) at each tested concentration. These metrics provide a quantitative evaluation of the reproducibility and variability in Tafel polarization parameters.

**Variable**	**1000 ppm**	**100 ppm**	**250 ppm**	**500 ppm**	**700 ppm**
n	3	3	3	3	3
$\bar{I}_{corr}$	40.4	101.0	82.0	74.7	57.7
SD$_{I_{corr}}$	3.44	26.9	5.03	1.79	13.6
SE$_{I_{corr}}$	1.99	15.5	2.91	1.03	7.85
t	4.30	4.30	4.30	4.30	4.30
IC-inf$_{I_{corr}}$	31.9	34.1	69.5	70.2	23.9
IC-sup$_{I_{corr}}$	48.9	168.0	94.5	79.1	91.5
CV%$_{I_{corr}}$	8.51	26.7	6.14	2.40	23.6
$\bar{E}_{corr}$	-420.0	-425.0	-428.0	-424.0	-420.0
SD$_{E_{corr}}$	6.03	8.39	1.15	7.02	1.15
SE$_{E_{corr}}$	3.48	4.84	0.667	4.06	0.667
IC-inf$_{E_{corr}}$	-435.0	-445.0	-431.0	-441.0	-423.0
IC-supp$_{E_{corr}}$	-405.0	-404.0	-425.0	-406.0	-417.0
CV%$_{E_{corr}}$	1.44	1.97	0.270	1.66	0.275

Table S7. Tafel summary statistics for extract W2, related to Figure 7b. Summary statistics include the mean corrosion current density ($\bar{I}_{\text{corr}}$) and corrosion potential ($\bar{E}_{\text{corr}}$), as well as the standard deviation (SD), standard error (SE), 95% confidence intervals (CI), and coefficient of variation (CV%) at each tested concentration. These metrics provide a quantitative evaluation of the reproducibility and variability in Tafel polarization parameters for W2.

**Variable**	**1000 ppm**	**100 ppm**	**250 ppm**	**500 ppm**	**700 ppm**
n	3	3	3	3	3
$\bar{I}_{corr}$	34.3	119.0	88.0	72.2	62.2
SD$_{I_{corr}}$	9.76	15.7	10.7	3.76	5.61
SE$_{I_{corr}}$	5.64	9.06	6.18	2.17	3.24
t	4.30	4.30	4.30	4.30	4.30
IC-inf$_{I_{corr}}$	10.1	79.7	61.4	62.8	48.3
IC-sup$_{I_{corr}}$	58.6	158.0	115.0	81.6	76.2
CV%$_{I_{corr}}$	28.4	13.2	12.2	5.21	9.02
$\bar{E}_{corr}$	-415.0	-426.0	-415.0	-412.0	-421.0
SD$_{E_{corr}}$	5.51	7.64	21.1	1.00	5.69
SE$_{E_{corr}}$	3.18	4.41	12.2	0.577	3.28
IC-inf$_{E_{corr}}$	-429.0	-445.0	-468.0	-414.0	-435.0
IC-supp$_{E_{corr}}$	-402.0	-407.0	-363.0	-410.0	-407.0
CV%$_{E_{corr}}$	1.33	1.79	5.09	0.243	1.35

Table S8. Tafel summary statistics for extract D1, related to Figure 7c. Summary statistics include the mean corrosion current density ($\bar{I}_{\mathrm{corr}}$) and corrosion potential ($\bar{E}_{\mathrm{corr}}$), as well as the standard deviation (SD), standard error (SE), 95% confidence intervals (CI), and coefficient of variation (CV%) at each tested concentration. These metrics provide a quantitative evaluation of the reproducibility and variability in Tafel polarization parameters for D1.

**Variable**	**1000 ppm**	**100 ppm**	**250 ppm**	**500 ppm**	**700 ppm**
n	3	3	3	3	3
$\bar{I}_{corr}$	65.7	316.0	151.0	104.0	99.4
SD$_{I_{corr}}$	18.3	42.5	16.8	20.8	18.3
SE$_{I_{corr}}$	10.6	24.5	9.71	12.0	10.6
t	4.30	4.30	4.30	4.30	4.30
IC-inf$_{I_{corr}}$	20.2	211.0	109.0	52.1	53.9
IC-sup$_{I_{corr}}$	111.0	422.0	193.0	155.0	145.0
CV%$_{I_{corr}}$	27.9	13.4	11.1	20.0	18.4
$\bar{E}_{corr}$	-409.0	-385.0	-394.0	-403.0	-406.0
SD$_{E_{corr}}$	2.08	21.0	11.4	12.7	4.16
SE$_{E_{corr}}$	1.20	12.1	6.57	7.36	2.40
IC-inf$_{E_{corr}}$	-415.0	-437.0	-422.0	-435.0	-416.0
IC-supp$_{E_{corr}}$	-404.0	-332.0	-365.0	-372.0	-395.0
CV%$_{E_{corr}}$	0.509	5.47	2.89	3.16	1.03

Table S9. Tafel summary statistics for extract D2, related to Figure 7d. Summary statistics include the mean corrosion current density ($\bar{I}_{\mathrm{corr}}$) and corrosion potential ($\bar{E}_{\mathrm{corr}}$), as well as the standard deviation (SD), standard error (SE), 95% confidence intervals (CI), and coefficient of variation (CV%) at each tested concentration. These metrics provide a quantitative evaluation of the reproducibility and variability in Tafel polarization parameters for D2.

**Variable**	**1000 ppm**	**100 ppm**	**250 ppm**	**500 ppm**	**700 ppm**
n	3	3	3	3	3
$\bar{I}_{corr}$	53.4	149.0	82.3	72.7	64.2
SD$_{I_{corr}}$	14.4	63.5	4.02	18.1	9.96
SE$_{I_{corr}}$	8.31	36.7	2.32	10.5	5.75
t	4.30	4.30	4.30	4.30	4.30
IC-inf$_{I_{corr}}$	17.6	-8.82	72.3	27.6	39.4
IC-sup$_{I_{corr}}$	89.1	307.0	92.3	118.0	88.9
CV%$_{I_{corr}}$	27.0	42.6	4.89	25.0	15.5
$\bar{E}_{corr}$	-424.0	-420.0	-429.0	-430.0	-425.0
SD$_{E_{corr}}$	2.65	24.1	9.24	6.11	3.51
SE$_{E_{corr}}$	1.53	13.9	5.33	3.53	2.03
IC-inf$_{E_{corr}}$	-431.0	-480.0	-452.0	-445.0	-433.0
IC-supp$_{E_{corr}}$	-417.0	-360.0	-406.0	-414.0	-416.0
CV%$_{E_{corr}}$	0.624	5.73	2.15	1.42	0.827

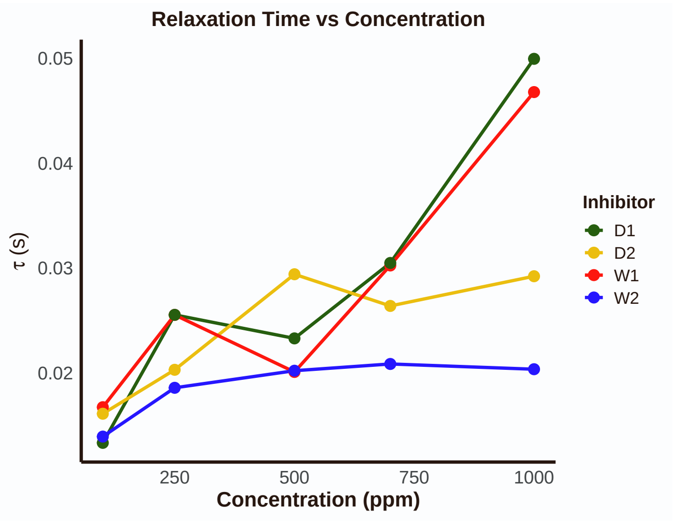

Figure S1. Relaxation time constant (τ) as a function of inhibitor concentration for each *Spirulina platensis* extract. Values of τ increased from the steel baseline (0.010 s) up to $\sim$0.047–0.050 s for W1 and D1 at 1000 ppm, suggesting the formation of more persistent interfacial films with slower discharge dynamics. In contrast, W2 and D2 plateaued at lower values ($\sim$0.02–0.03 s), indicative of weaker or less stable film buildup. Since τ is rarely reported in studies of microalgal inhibitors, these results provide novel insights into the time-dependent protective behavior of green corrosion inhibitors.

Table S10. Tukey post hoc grouping of *Spirulina platensis* extracts at different concentrations, related to Figure 6a. Treatments are stratified into statistically distinct resistivity tiers based on polarization resistance (R_p). D1 at 1000 ppm achieved the highest resistivity classification ("Very High"), while orbital-shaken extracts (W2) consistently occupied the lower tiers ("Low" or "Low–moderate") across the tested concentration range. This statistical classification highlights the influence of biomass state and extraction method on inhibitor performance.

**Resistivity Level**	**Group(s)**	**Interpretation**
Very High Resistivity	D1-1000ppm	Highest inhibition performance; significantly superior to all other treatments.
High Resistivity	D2-1000ppm	Strong performance; statistically lower than D1_1000ppm but higher than all others.
Moderately High	W1-1000ppm, D2-700ppm	Comparable to each other; significantly better than lower tiers.
Moderate	D1-700ppm, W1-700ppm, D1-500ppm, D2-500ppm, D1-250ppm	Mid-range inhibition; significant differences from top groups.
Low to Moderate	W2-1000ppm, W1-500ppm, W2-700ppm, D2-250ppm	Lower performance; not significantly different among themselves.
Low	D2-100ppm, W2-500ppm, W1-250ppm, W1-100ppm	Among the lowest effective concentrations; still significantly better than Steel.
Very Low	D1-100ppm, W2-250ppm	Minimal improvement over baseline.
Baseline	Steel	Control group; significantly lower than all inhibitor treatments.

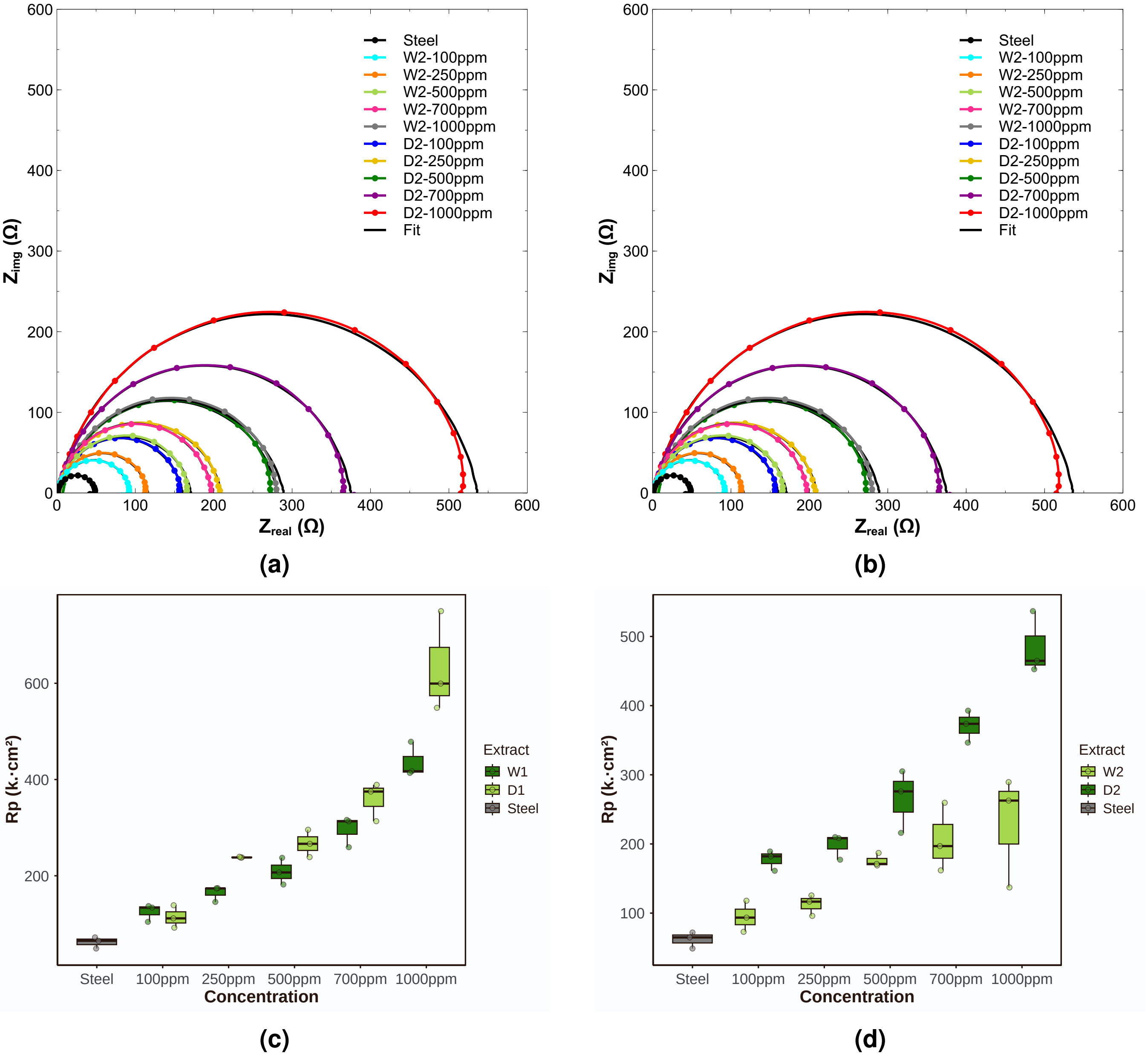

Figure S2. Comparison of Nyquist responses and charge-transfer resistance distributions for wet and dry biomass extracts, related to Figure 4 (a) Nyquist plots for W1 vs. D1 across all concentrations, and (b) Nyquist plots for W2 vs. D2 across all concentrations, illustrating the influence of extraction method and biomass state on interfacial impedance behavior. (c–d) Box-plot summaries of the corresponding charge-transfer resistance (R_{ct}) distributions highlight consistently higher R_{ct} values for ultrasound-assisted extracts (W1, D1) compared to orbital-shaken counterparts (W2, D2), with the dry–ultrasound combination (D1) yielding the most resistive response. Boxes represent the interquartile range (IQR), center lines indicate the median, and whiskers denote minimum and maximum values (n = 3 per condition).

Table S11. Summary of corrosion rate (CR) and inhibition efficiency (EI%) for *Spirulina platensis* extracts, related to Figure 9. This table reports the mean corrosion rate and inhibition efficiency values expressed as mean ± standard deviation ($n = 3$), along with performance tiers derived from Fisher's Least Significant Difference (LSD) test at $\alpha = 0.05$. The classification stratifies treatments into distinct performance groups: D1 exhibited the highest inhibition efficiency and lowest corrosion rate ("High"), followed by D2 in the "High–Moderate" category, whereas W1 and W2 occupied the "Moderate" tier. The uninhibited steel (Blank) consistently presented the highest corrosion rate, confirming the protective effect of all extracts.

Sample	CR (mm/year)	EI (%)	LSD-based Group	Performance Category
Steel	11.16 ± 0.92	–	a (CR)	None (Control)
W2	2.57 ± 0.35	76.99 ± 3.12	b (CR), c (EI)	Moderate
W1	2.44 ± 0.08	78.19 ± 0.70	b (CR), c (EI)	Moderate
D2	1.77 ± 0.08	84.10 ± 0.70	bc (CR), b (EI)	High–Moderate
D1	1.41 ± 0.06	87.34 ± 0.58	c (CR), a (EI)	High

LSD value (CR): 0.806 *LSD value (EI%):* 3.13

Table S12. Life Cycle Thinking (LCT) assessment of Spirulina-derived corrosion inhibitors. Summary of key environmental and functional outcomes across the four evaluated life cycle stages.

Life cycle stage	Aspects evaluated	Key findings	Environmental implication	Ref.
**(i) Biomass production**	CO_2 fixation, water and energy use, renewable origin	Protein-, polysaccharide-, and phenolic-rich biomass; estimated CO_2 capture of 1.6–2.2 kg per kg of dry biomass; moderate water and energy inputs	Renewable and biodegradable raw material; favorable upstream impact	10,11
**(ii) Extraction using ultrasound and orbital agitation with methanol**	Extraction energy, solvent consumption, process efficiency	Ultrasound-assisted method (D1) showed higher extraction efficiency with lower solvent demand; orbital agitation required higher volumes; methanol recovery 80–90%	Moderate impact due to methanol use, mitigated by high recovery and low environmental persistence	12,13
**(iii) Use phase: corrosion inhibition performance**	Inhibition efficiency, stability over time, corrosion rate	D1: 90–92% initial efficiency and 77% after 72 h; corrosion rate reduced to 1.41 mm·year^{-1} at 1000 ppm; other extracts showed moderate protection	High functional benefit: lower corrosion reduces replacement frequency, material waste, and long-term energy demand	14
**(iv) End of life: biodegradability and waste handling**	Residue composition, biodegradability, methanol disposal	Microalgal extract is biodegradable; residual solution contains methanol and must be treated as non-aqueous organic waste	Lower impact than synthetic inhibitors; solvent requires proper handling but does not generate persistent or toxic residues	15

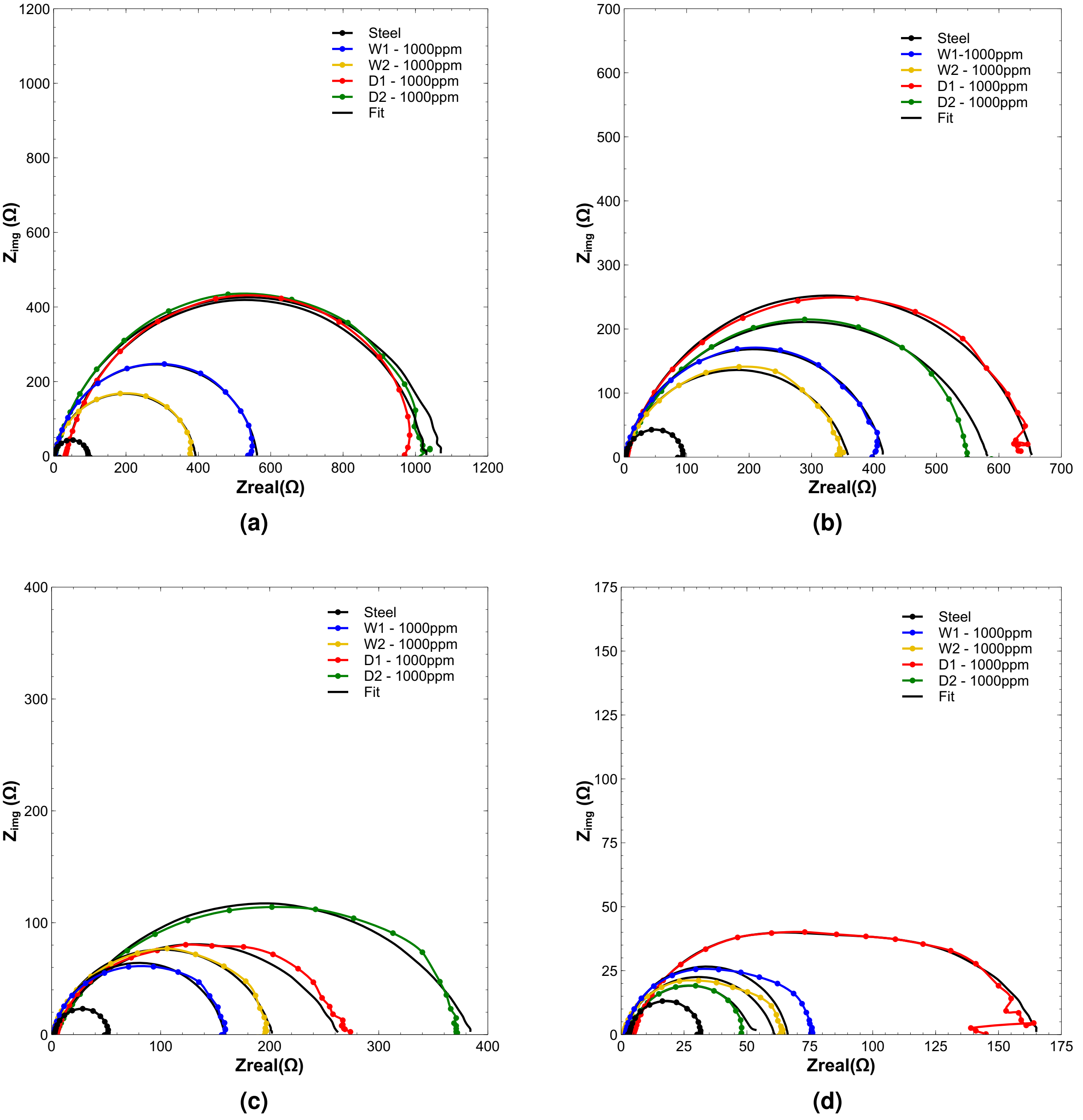

Figure S3. Time-dependent Nyquist plots of carbon steel in 1 M HCl with *Spirulina platensis* extracts (1000 ppm), related to Figure 12. (a) 3 h, (b) 24 h, (c) 48 h, and (d) 72 h immersion. The evolution of the semicircle diameters reflects changes in charge-transfer resistance (R_{ct}) over time. Rapid early adsorption is observed at 3 h, followed by relative stability at 24 h, moderate film degradation at 48 h, and a more pronounced decline by 72 h. Among the extracts, dry–ultrasound treatment (D1) maintained the highest and most persistent interfacial resistance, whereas W2 exhibited the weakest protective behavior.

References

1. Çelekli, A., Yavuzatmaca, M., and Bozkurt, H. (2009). An eco-friendly process: Predictive modelling of copper adsorption from aqueous solution on *spirulina platensis*. Journal of Hazardous Materials *173*, 123–129. doi: 10.1016/j.jhazmat.2009.08.057.

2. El-Shazoly, R.M., Yousef, S., Hifney, A.F. et al. (2025). Effectiveness of algae as a low-cost alternative input to stimulate *sesamum indicum* l. growth and productivity for sustainable purposes. Journal of Soil Science and Plant Nutrition. URL: https://doi.org/10.1007/s42729-025-02651-1. doi: 10.1007/s42729-025-02651-1. Published online: August 11, 2025.

3. Arora, R., Sudhakar, K., and Rana, R.S. (2021). Biochemical and thermal analysis of spirulina biomass through ftir. Energy Engineering *118*, 1045–1056. doi: 10.32604/EE.2021.016082.

4. Cirulis, J.T., Scott, J.A., and Ross, G.M. (2013). Management of oxidative stress by microalgae. Canadian Journal of Physiology and Pharmacology *91*, 15–21.

5. Adarm, P., and Kumari, P. (2017). Ftir characterization of *spirulina platensis* biomolecules. International Journal of Biological Macromolecules *102*, 37–45.

6. Liu, J., Zhu, C., Li, Z., and Zhou, H. (2022). Screening of spirulina strains for high copper adsorption capacity through fourier transform infrared spectroscopy. Frontiers in Microbiology *13*, 952597. URL: https://doi.org/10.3389/fmicb.2022.952597. doi: 10.3389/fmicb.2022.952597.

7. Astolfi, A.L., Rempel, A., Cavanhi, V.A.F., Alves, M., Deamici, K.M., Colla, L.M., and Costa, J.A.V. (2019). Simultaneous saccharification and fermentation of *spirulina* sp. and corn starch for the production of bioethanol and obtaining biopeptides with high antioxidant activity. Bioresource Technology *294*, 122698. URL: https://doi.org/10.1016/j.biortech.2019.122698. doi: 10.1016/j.biortech.2019.122698.

8. Bikmurzin, R., Bandzevičiūtė, R., Maršalka, A., Maneikis, A., and Kalėdienė, L. (2022). Ft-ir method limitations for β-glucan analysis. Molecules *27*, 4616. URL: https://doi.org/10.3390/molecules27144616. doi: 10.3390/molecules27144616.

9. Parihar, A., Vongsvivut, J., and Bhattacharya, S. (2019). Synchrotron-based infra-red spectroscopic insights on thermo-catalytic conversion of cellulosic feedstock to levoglucosenone and furans. ACS Omega *4*, 8747–8757. URL: https://doi.org/10.1021/acsomega.8b03681. doi: 10.1021/acsomega.8b03681.

10. Miranda, A.M., Hernandez-Tenorio, F., Ocampo, D., Vargas, G.J., and Sáez, A.A. (2022). Trends on co2 capture with microalgae: A bibliometric analysis. Molecules *27*, 4669. doi: 10.3390/molecules27154669.

11. Cheirsilp, B., Maneechote, W., Srinuanpan, S., and Angelidaki, I. (2023). Microalgae as tools for bio-circular-green economy: Zero-waste approaches for sustainable production and biorefineries of microalgal biomass. Bioresource Technology *387*, 129620. doi: 10.1016/j.biortech.2023.129620.

12. Liu, Y., Liu, X., Cui, Y., and Yuan, W. (2022). Ultrasound for microalgal cell disruption and product extraction: A review. Ultrasonics Sonochemistry *87*, 106054. doi: 10.1016/j.ultsonch.2022.106054.

13. De Bhowmick, G., Plouviez, M., Reis, M.G., Guieysse, B., Everett, D.W., Agnew, M.P., Maclean, P., and Thum, C. (2024). Evaluation of extraction techniques for recovery of microalgal lipids under different growth conditions. ACS Omega *9*, 27976–27986. doi: 10.1021/acsomega.4c00221.

14. Almanza, E., Gutierrez Pua, L.d.C., Pineda, Y., Rozo, W., Marquez, M., and Fonseca, A. (2024). Eco-friendly Chlorella vulgaris extracts for corrosion protection of steel in acidic environments. Heliyon *10*, e39717. doi: 10.1016/j.heliyon.2024.e39717.

15. Baskar, P., Annadurai, S., Panneerselvam, S., Prabakaran, M., and Kim, J. (2023). An outline of employing metals and alloys in corrosive settings with ecologically acceptable corrosion inhibitors. Surfaces *6*, 380–409. doi: 10.3390/surfaces6040027.
